# Podocyte mPGES‐2 Determines Renal Aging and Contributes to Senile Osteoporosis

**DOI:** 10.1111/acel.70609

**Published:** 2026-06-25

**Authors:** Dandan Zhong, Chang Hao, Mengyue Li, Jing Liu, Zheng Xu, Jianteng Zhou, Lu Zhao, Siyu Ni, Zhenchao Hu, Yue Sun, Yingying Zou, Dong Sun, Hao Guo, Zhanjun Jia, Dong Guo, Jun‐Li Cao, Ying Sun

**Affiliations:** ^1^ Jiangsu Key Laboratory of Geriatric Precision Medicine and Aging Intervention Xuzhou Medical University Xuzhou Jiangsu China; ^2^ Jiangsu Key Laboratory of New Drug Research and Clinical Pharmacy Xuzhou Medical University Xuzhou Jiangsu China; ^3^ Taicang Loujiang New Town Hospital Suzhou Jiangsu China; ^4^ Jiangsu Province Key Laboratory of Anesthesiology & Jiangsu Province Key Laboratory of Anesthesia and Analgesia Application Technology & NMPA Key Laboratory for Research and Evaluation of Narcotic and Psychotropic Drugs Xuzhou Medical University Xuzhou Jiangsu China; ^5^ Jiangsu Engineering Center for Precision Diagnosis and Treatment Research of Polygenic Diseases, Key Laboratory of Genetic Foundation and Clinical Application, Department of Genetics Xuzhou Medical University Xuzhou China; ^6^ Nanjing Key Laboratory of Pediatrics Children's Hospital of Nanjing Medical University Nanjing Jiangsu China; ^7^ Department of Nephrology Affiliated Hospital of Xuzhou Medical University Xuzhou Jiangsu China; ^8^ Clinical Research Center for Kidney Disease Xuzhou Medical University Xuzhou Jiangsu China; ^9^ Department of Science and Technology Xuzhou Medical University Xuzhou Jiangsu China

**Keywords:** kidney–bone axis, mPGES‐2, osteoporosis, podocyte, renal aging

## Abstract

Renal aging shortens healthspan and propagates organ dysfunction beyond the kidney, yet its molecular drivers remain incompletely defined. Here we identify microsomal prostaglandin E synthase‐2 (mPGES‐2) as a critical regulator of renal aging and its skeletal consequence. Genetic ablation of *Ptges2* improved health indices in aged mice, prolonged median survival, and markedly alleviated glomerulosclerosis, podocyte injury, and renal senescence. Single‐cell transcriptomic analysis, together with podocyte‐ and tubule‐specific knockout models, showed that podocyte mPGES‐2, rather than tubular mPGES‐2, is the dominant intrarenal driver of aging‐related kidney injury. Mechanistically, mPGES‐2 promoted podocyte senescence through a PGE_2_/EP1 signaling axis. Podocyte‐specific *Ptges2* deletion also mitigated age‐related osteoporosis and restored renal calcitriol and α‐klotho, supporting a kidney‐bone mechanism secondary to impaired renal endocrine function. Consistent with the genetic models, pharmacological inhibition of mPGES‐2 with SZ0232 attenuated renal aging and improved bone microarchitecture in aged mice. Both genetic deficiency and pharmacological inhibition of mPGES‐2 were well tolerated, with no overt adverse effects on major organs. These findings identify podocyte mPGES‐2 as a druggable determinant of renal aging and a potential therapeutic target for aging‐associated osteoporosis.

## Graphical abstract

Podocyte mPGES‐2 drives renal aging through PGE_2_/EP1‐dependent senescence and disrupts renal endocrine support for bone homeostasis. Genetic deletion or pharmacological inhibition of mPGES‐2 protects aged mice from kidney dysfunction and osteoporosis.

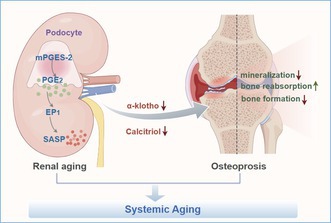

## 1 Introduction

Aging is a systemic process shaped by progressive changes within individual tissues and by crosstalk among organs (Ferrucci et al. 2020; Kumar et al. 2024; Schaum et al. 2020; Wagner et al. 2023). Although multiple conceptual frameworks have been proposed, no single model fully explains how aging unfolds across organs with distinct regenerative capacities and functional demands (Campisi et al. 2019; Cohen et al. 2020). This heterogeneity is particularly relevant at the cellular level, where certain cell types deteriorate earlier or more severely than others (Ahadi et al. 2020; Tuttle et al. 2020). In parallel, dysfunction in one organ can accelerate decline in others, thereby promoting multimorbidity in late life (Elliott et al. 2021; Mao et al. 2024). Defining the molecular basis of these inter‐organ interactions is therefore central to the development of effective geroprotective strategies.

The kidneys are among the organs most susceptible to aging, in part because of the limited self‐repair capacity of nephrons (Benzing and Schumacher 2023). Aged kidneys undergo characteristic structural changes, including glomerulosclerosis, podocyte loss, tubular injury, and interstitial fibrosis, which collectively impair filtration, endocrine function, and tissue homeostasis (Fang et al. 2020). These alterations increase susceptibility to additional insults and may propagate dysfunction beyond the kidney. Despite the clinical importance of renal aging, the upstream drivers of these changes, the cell types that initiate them, and the extent to which they can be therapeutically modified remain incompletely understood.

The kidney is also a major regulator of mineral metabolism and skeletal homeostasis. Through calcitriol production, α‐klotho expression, and coordinated handling of calcium and phosphate, the kidney exerts broad control over bone remodeling (Agoro and White 2023; Hruska et al. 2017). With aging, bone strength declines, trabecular architecture deteriorates, and mineral balance becomes increasingly fragile. Although kidney‐bone communication is well recognized, the molecular mechanisms by which renal aging contributes to skeletal deterioration remain poorly defined. Clarifying this axis may help explain why osteoporosis frequently accompanies age‐related kidney dysfunction.

Chronic inflammation, also termed inflammaging, is a pervasive feature of advanced age and contributes to multi organ damage, age related disease, and increased mortality (Park et al. 2024). Prostaglandin E_2_ (PGE_2_), a central lipid mediator of inflammation derived from arachidonic acid metabolism, is produced in many tissues, with the kidney representing a major source (Loynes et al. 2018). PGE_2_ levels rise with aging and have been linked to chronic inflammation, metabolic dysfunction, and cognitive decline (Geurts et al. 2024; Minhas et al. 2021). However, the mechanisms governing PGE_2_ production and signaling during physiologic aging remain insufficiently understood.

Microsomal prostaglandin E synthase‐2 (mPGES‐2), encoded by *Ptges2*, is one of the three known prostaglandin E synthases (PGESs) that directly affect the biosynthesis of PGE_2_. mPGES‐2 is constitutively expressed under normal physiological conditions, suggesting its role in maintaining tissue homeostasis. Unlike the inducible mPGES‐1, mPGES‐2 is constitutively expressed in many tissues and has been implicated in the maintenance of cellular homeostasis. Importantly, mPGES‐2 is a dual‐function enzyme; under most in vivo conditions it preferentially generates malondialdehyde (MDA) rather than PGE_2_ (Yamada and Takusagawa 2007). Prior work, including ours, has implicated mPGES‐2 in metabolic disease and tissue injury (Lindner et al. 2007; Nitz et al. 2007; Sun et al. 2014; Zhong et al. 2022), but its role in natural aging has not been defined. Whether mPGES‐2 contributes to renal aging, and if so through which cell type and downstream pathway, remains unknown.

In this study, we show that mPGES‐2 is progressively upregulated in aged kidneys in both humans and mice and that its genetic deletion substantially attenuates renal aging. By integrating single‐cell transcriptomics with conditional mouse models, we identify podocytes as the principal intrarenal compartment through which mPGES‐2 drives renal injury. Mechanistically, mPGES‐2 promotes podocyte senescence through a PGE_2_/EP1 signaling pathway and impairs the renal endocrine milieu associated with bone homeostasis. Accordingly, podocyte‐specific *Ptges2* deletion and pharmacological inhibition with the selective mPGES‐2 inhibitor SZ0232 both ameliorate age‐related osteoporosis. These findings position podocyte mPGES‐2 as a tractable target for intervention in renal aging and its systemic skeletal complication.

## 2 Results

### 2.1 mPGES‐2 Deficiency Promotes Longevity and Improves Health

**FIGURE 1:**
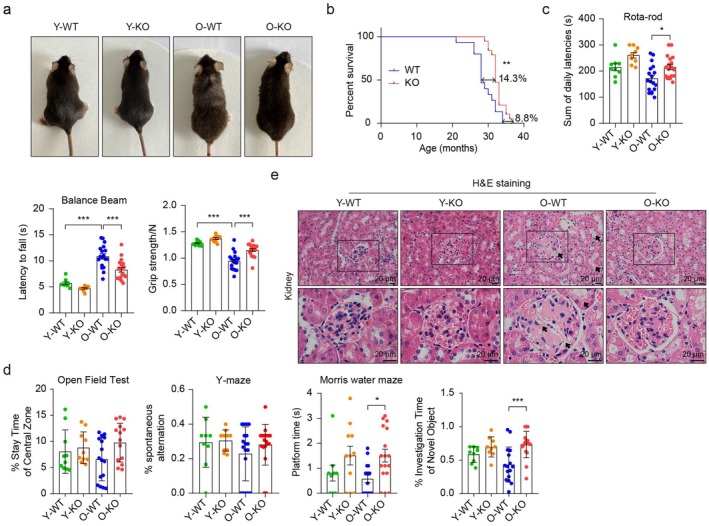
mPGES‐2 deficiency benefits longevity and supports overall health. (a) Representative images of mPGES‐2 wild‐type (WT) and knockout (KO) mice at young (8 weeks) and old ages (2 years). (b) Lifespan of mPGES‐2 WT and KO mice. (c) Motor activity evaluated by Rota‐rod, balance beam, and grip strength tests. (d) Learning and memory tests analyzed by open field, Y‐maze, Morris water maze and novel object recognition test. (e) Representative images showing (hematoxylin and eosin) H&E staining of kidney specimens. Data are presented as mean ± SEM. *p < 0.05, **p < 0.01, and ***p < 0.001; Survival was compared by the log rank (Mantel–Cox) test and one‐way ANOVA with Tukey's test for (c) and (d).

To examine the impact of mPGES‐2 on organismal aging, we compared young (8‐week‐old) and aged (2‐year‐old) wild‐type and *Ptges2*‐knockout mice. No obvious phenotypic differences were detected between young wild‐type and knockout animals (Figure 1a). In contrast, aged *Ptges2*‐knockout mice appeared healthier than age‐matched controls, with smoother coats and improved overall appearance, and they exhibited prolonged survival (Figure 1a,b). *Ptges2* deletion also improved motor performance and cognitive readouts in aged mice (Figure 1c,d). We next surveyed tissues commonly affected by aging, including kidney, bone, brain, muscle, fat, and liver. Protective effects were observed across several organs, but the most striking improvement was in the kidney, where age‐associated structural deterioration, particularly glomerular injury, was markedly attenuated (Figure 1e; Figure S1a, Appendix S1). Serum ALT, AST, BUN, and creatinine remained within physiological reference ranges and were lower in aged knockout than in age‐matched wild‐type controls (Figure S1b, Appendix S1), indicating that mPGES‐2 deficiency is well tolerated and preserves age‐related biochemical homeostasis. These data suggest that loss of mPGES‐2 improves late‐life health status, with especially prominent benefits in the kidney.

### 2.2 Global *Ptges2* Knockout Attenuates Renal Aging and Kidney Injury in Aged Mice

**FIGURE 2:**
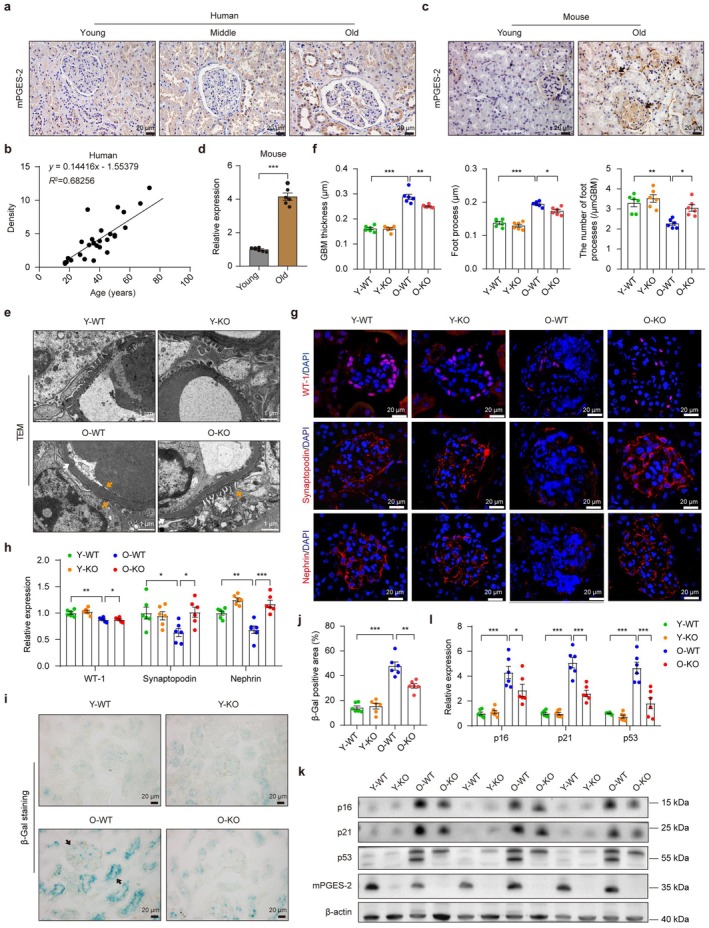
*Ptges2* knockout mitigates renal aging and related kidney dysfunction. (a) Representative images of mPGES‐2 expression in human kidneys, scale bars = 20 μm. (b) Correlation analysis of mPGES‐2 protein expression with age in human kidneys. (c) Expression of mPGES‐2 in young and old mouse kidneys, scale bars = 20 μm. (d) Quantification of mPGES‐2 expression in mouse kidneys. (e) Representative images of transmission electron microscopy (TEM). (f) Measurement of glomerular basement membrane (GBM) thickness, foot process length, and number of foot processes by TEM. (g) Immunofluorescence (IF) staining of WT‐1, synaptopodin and nephrin, podocyte markers. (h) Quantification of podocyte markers. (i) Representative images of β‐Gal staining. (j) Quantification of positive β‐Gal staining. (k) Representative blots of p16, p21, p53, key aging biomarkers. (l) Quantification of aging biomarkers. Data are presented as mean ± SEM. *p < 0.05, **p < 0.01, and ***p < 0.001; unpaired Student's *t*‐test was used for (d) and one‐way ANOVA with Tukey's test for f, h, j, and l.

Given the pronounced renal benefit observed in aged *Ptges2*‐knockout mice, we next examined the role of mPGES‐2 in renal aging in greater detail. In human kidney tissue, mPGES‐2 expression increased progressively with age and showed a significant positive correlation with donor age (Figure 2a,b). A similar age‐dependent increase was observed in mouse kidneys, with prominent staining in both glomerular and tubulointerstitial compartments (Figure 2c,d). Functionally, *Ptges2* deletion reduced the urinary albumin‐to‐creatinine ratio, increased glomerular filtration rate (GFR), and limited mesangial matrix expansion in aged mice (Figure S2a–d, Appendix S1). Transmission electron microscopy further showed reduced podocyte loss and improved ultrastructural organization after *Ptges2* knockout (Figure 2e,f). Consistent with these findings, immunofluorescence demonstrated preservation of the podocyte markers WT‐1, synaptopodin, and nephrin in aged knockout kidneys (Figure 2g,h). *Ptges2* deletion also reduced tubular cell death and renal fibrosis (Figure S2e–j, Appendix S1). At the level of cellular senescence, kidneys from aged knockout mice displayed reduced SA‐β‐gal staining and lower expression of p16, p21, p53, and senescence‐associated secretory phenotype (SASP) factors (Figure 2i–l; Figure S2k, Appendix S1). Together, these data show that mPGES‐2 contributes to structural, functional, and molecular features of renal aging.

### 2.3 Single‐Cell Transcriptomic Analysis Reveals Podocyte‐Enriched *Ptges2* Expression in Aged Glomeruli

**FIGURE 3:**
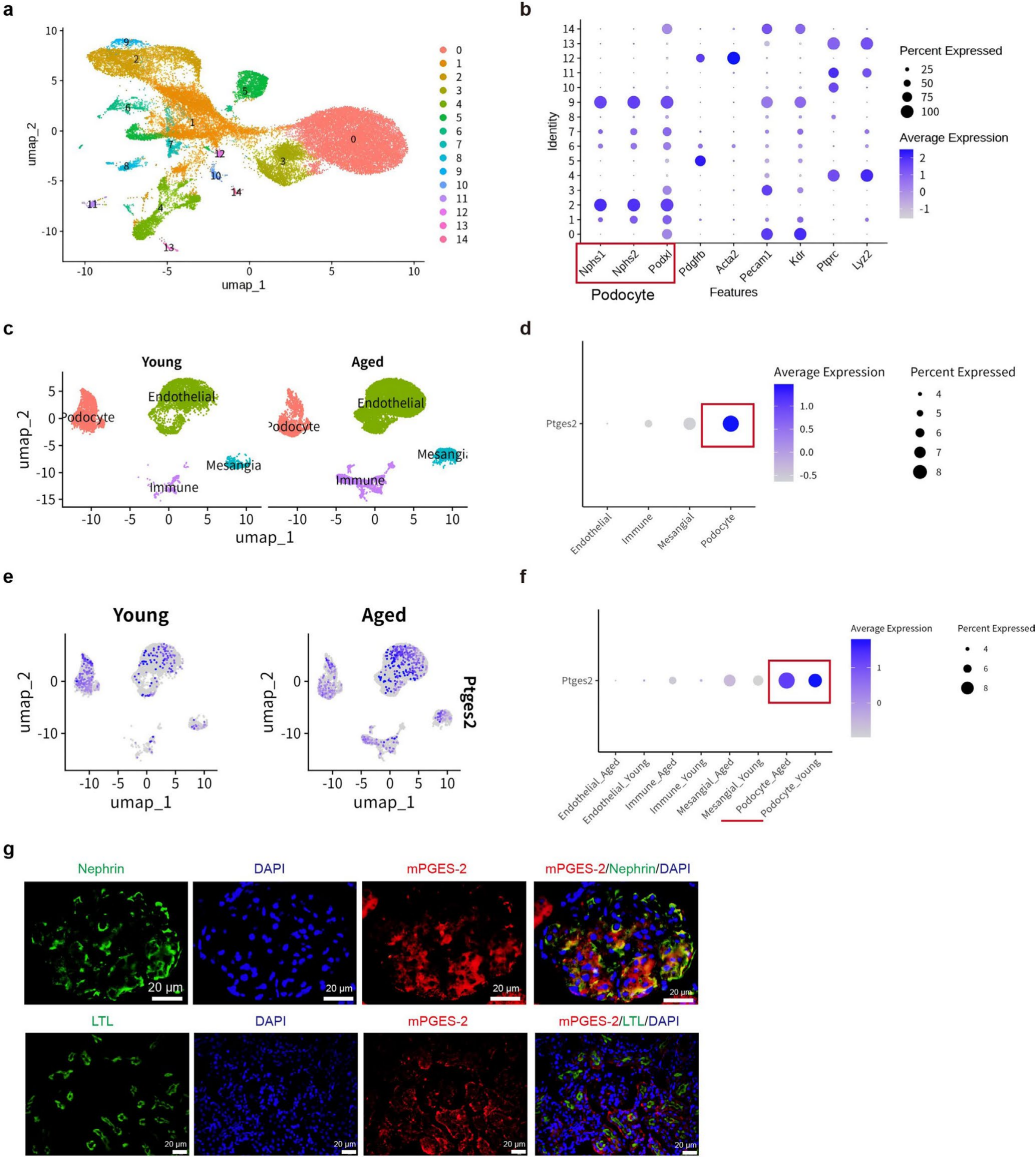
Single‐cell transcriptomic analysis reveals a podocyte‐enriched expression pattern of *Ptges2* in aged glomeruli. (a) UMAP of 15 unsupervised clusters from GSE240374 mouse kidney. (b) Dot plot of canonical marker genes used for cell‐type annotation. Podocyte clusters were identified by *Nphs1*, *Nphs2*, and *Podxl*; mesangial cells by *Pdgfrb* and *Acta2*; endothelial cells by *Pecam1* and *Kdr*; and immune cells by *Ptprc* and *Lyz2*. Dot size indicates the percentage of cells expressing the indicated gene, and color indicates average expression level. (c) UMAP plots showing the distribution of major glomerular cell populations in young and aged kidneys, including podocytes, endothelial cells, mesangial cells, and immune cells. (d) Dot plot showing *Ptges2* expression across annotated glomerular cell populations. *Ptges2* expression was enriched in podocytes relative to the other glomerular cell types. (e) Feature plots showing the distribution of *Ptges2* expression in glomerular cells from young and aged kidneys. (f) Dot plot showing *Ptges2* expression across glomerular cell populations stratified by age. Compared with young podocytes, aged podocytes showed higher *Ptges2* expression and a greater proportion of *Ptges2*‐expressing cells. (g) Dual label immunofluorescence in mouse kidney sections. Top row: mPGES‐2 with Nephrin (podocyte marker). Bottom row: mPGES‐2 with LTL (proximal tubule marker). Scale bars 20 μm.

Because global *Ptges2* deletion markedly improved glomerulosclerosis and podocyte injury, we next sought to define the glomerular cell types in which *Ptges2* is expressed. We therefore analyzed single‐cell transcriptomic data (GSE240374) from glomerular cells isolated from young and aged kidneys. Unsupervised clustering identified distinct glomerular cell populations, which were annotated using canonical marker genes, including *Nphs1*, *Nphs2*, and *Podxl* for podocytes, *Pdgfrb* and *Acta2* for mesangial cells, *Pecam1* and *Kdr* for endothelial cells, and *Ptprc* and *Lyz2* for immune cells (Figure 3a,b). Among these populations, *Ptges2* showed a podocyte‐enriched expression pattern and the proportion of *Ptges2* ^+^ cells was higher in aged than in young podocytes, indicating an age‐dependent shift (Figure 3c–f). We next examined the spatial localization of mPGES‐2 by dual label immunofluorescence in structurally preserved mouse kidney sections. mPGES‐2 immunoreactivity was predominantly detected in nephrin‐positive podocytes within the glomerulus and was additionally observed in *Lotus tetragonolobus* lectin (LTL)‐positive proximal tubular cells, albeit at a relatively lower intensity (Figure 3g). Taken together, these findings support a podocyte enriched, but not podocyte exclusive, localization of mPGES‐2 within the kidney.

### 2.4 Podocyte mPGES‐2 Determines Renal Aging and Associated Dysfunction

**FIGURE 4:**
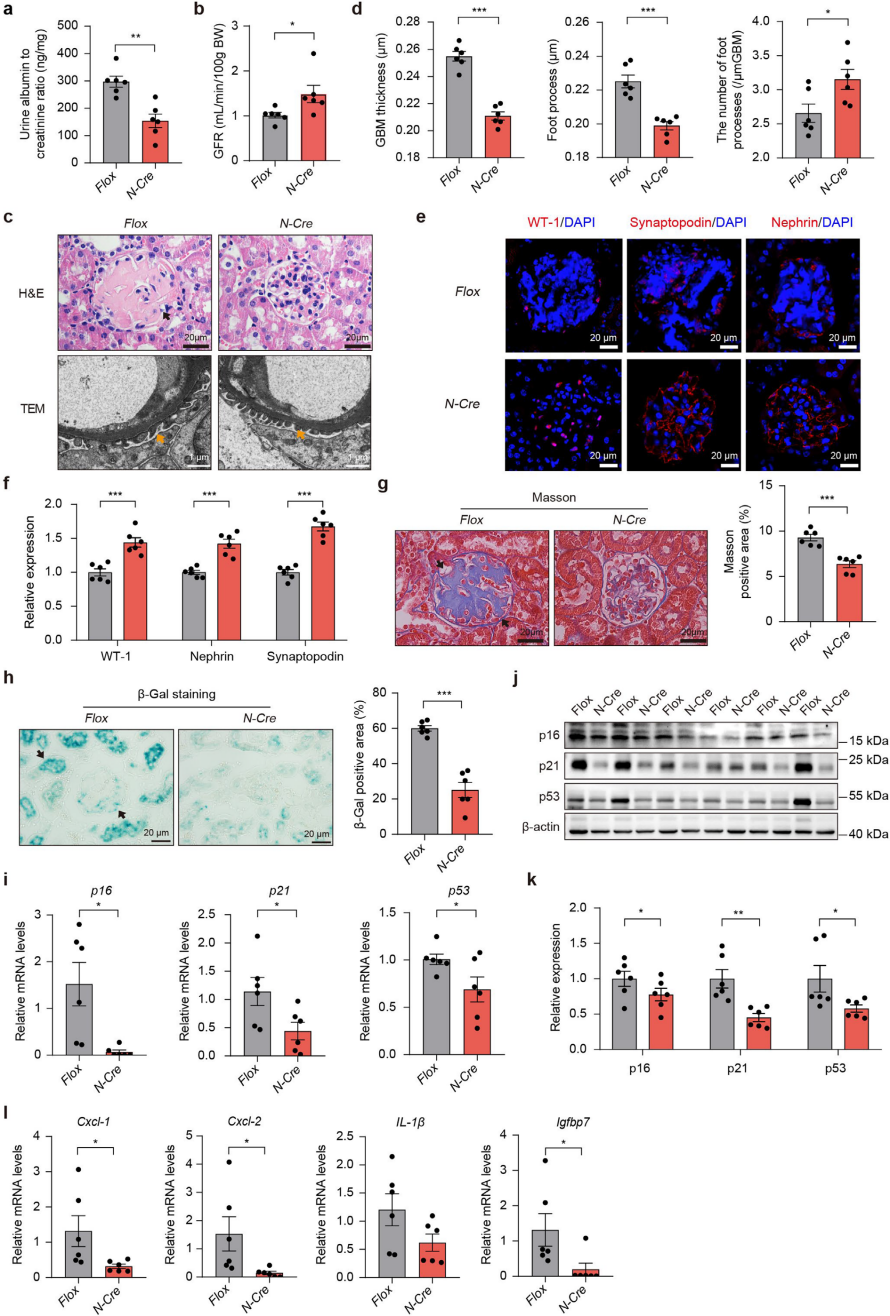
Podocyte‐specific *Ptges2* deletion ameliorates kidney injury and renal aging. Podocyte‐specific *Ptges2* knockout mice were aged naturally and sacrificed at 2 years of age for the following assays: (a) Urine albumin‐to‐creatinine ratio. (b) Glomerular filtration rate (GFR). (c) Assessment of morphological changes by H&E staining and TEM assay. (d) Quantification of GBM thickness, foot process length, and number of foot processes by TEM. (e) Podocyte markers in the glomeruli. (f) Quantification of podocyte markers. (g) Renal fibrosis analyzed by Masson's trichrome staining. (h) Renal aging evaluated by β‐Gal staining. (i) mRNA levels of *p16*, *p21*, and *p53*. (j) Representative blots of aging biomarkers. (k) Quantification of aging biomarkers. (l) Senescence‐associated secretory phenotype (SASP) factors levels. *N‐Cre*: *Ptges2* ^*f/f*^ *;Nphs2‐Cre*. *Flox*: *Ptges2* ^*f/f*^. Data are presented as mean ± SEM. *p < 0.05, **p < 0.01, and ***p < 0.001; unpaired Student's *t*‐test was used for statistical analysis.

In line with the single‐cell transcriptomic data showing a podocyte‐enriched expression pattern of *Ptges2* in aged glomeruli, and considering the crucial role of podocytes in maintaining the glomerular barrier and filtration function (Nagata 2016), we generated podocyte‐specific *Ptges2*‐knockout mice to further test whether podocyte mPGES‐2 is functionally required for renal aging. In aged mice, podocyte‐specific *Ptges2* deletion significantly lowered the urinary albumin‐to‐creatinine ratio and increased glomerular filtration rate (Figure 4a,b). Glomerulosclerosis, podocyte damage, and renal fibrosis were also attenuated (Figure 4c–g; Figure S3a,b, Appendix S1). Importantly, podocyte‐specific *Ptges2* knockout also reduced renal aging, as demonstrated by reduced SA β galactosidase (β‐Gal) positivity and lower expression of aging biomarkers (Figure 4h–l). These results indicate that podocyte mPGES‐2 is a major determinant of aging‐related kidney injury.

### 2.5 Tubule‐Specific *Ptges2*‐Knockout Weakly Ameliorates Renal Aging and Related Symptoms

Given that mPGES‐2 was also detected in renal tubular cells and that global *Ptges2* deletion ameliorated tubular injury, including tubular dilation, brush border loss, and apoptosis, we next asked whether tubular mPGES‐2 makes an independent contribution to renal aging. To answer it, we generated tubule‐specific *Ptges2*‐knockout mice. Tubule‐specific deletion produced only partial improvement in renal aging and associated kidney injury (Figure S4a–j, Appendix S1), and the magnitude of protection was clearly less than that observed in podocyte‐specific knockout mice, which largely phenocopied the global knockout. These findings suggest that tubular mPGES‐2 contributes to renal aging, but to a lesser extent than podocyte mPGES‐2.

### 2.6 mPGES‐2 Accelerates Renal Aging via the PGE_2_ /EP1 Signaling Pathway

**FIGURE 5:**
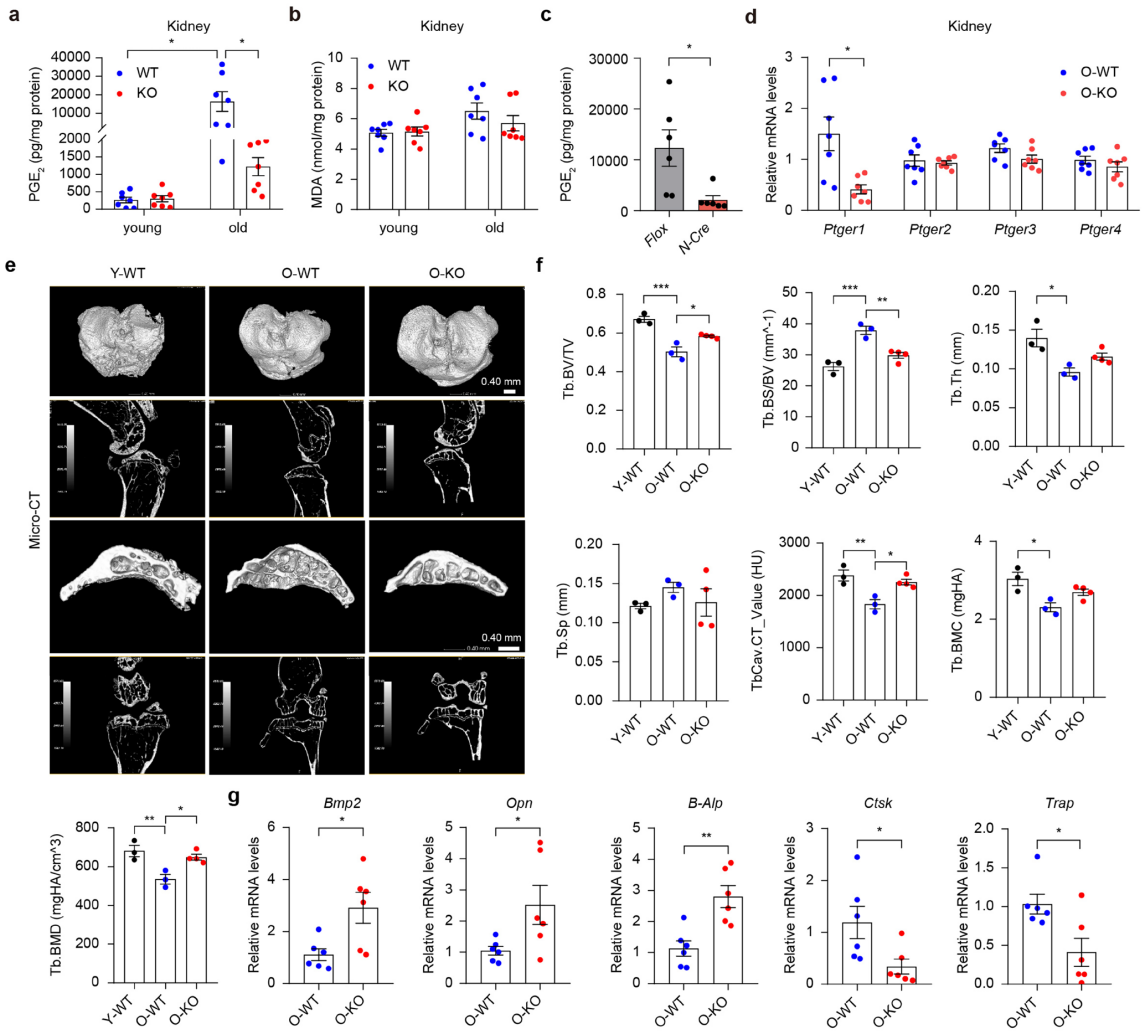
Effect of global *Ptges2* knockout on bone formation and health. (a) Effect of *Ptges2* knockout on the content of PGE_2_ in the kidney. (b) Effect of *Ptges2* knockout on the content of MDA in the kidney. (c) Effect of podocyte *Ptges2* specific knockout on the content of PGE_2_ in the kidneys. (d) *Ptges2* knockout on expression of four EP receptors. (e) Effect of *Ptges2* knockout on bone microarchitecture by micro‐computed tomography (μCT). (f) Quantified indices by μCT. (g) *Ptges2* knockout on the expression of osteoblast and osteoclast biomarkers. Data are presented as mean ± SEM. *p < 0.05, **p < 0.01, and ***p < 0.001; unpaired Student's t‐test was used for c, d, g and one‐way ANOVA with Tukey's test was used for a, b, f.

mPGES‐2 was initially identified as a PGE_2_ synthase, but it was later recognized as a dual‐function enzyme that preferentially generates MDA rather than PGE_2_ under most in vivo conditions (Yamada and Takusagawa 2007). Consistent with this view, several studies, including ours, have shown that *Ptges2* manipulation does not measurably alter renal PGE_2_ synthesis in a number of pathological settings (Jania et al. 2009; Yamada and Takusagawa 2007; Zhong et al. 2024; Zhong, Quan, et al. 2023). However, whether mPGES‐2 contributes to renal aging through PGE_2_‐related signaling remains unclear. To address this, we measured the levels of its two metabolites, PGE_2_ and MDA. Unexpectedly, global *Ptges2* knockout did not affect MDA content but significantly reduced PGE_2_ levels in aged mice (Figure 5a,b). Likewise, podocyte‐specific *Ptges2* silencing reduced renal PGE_2_ content (Figure 5c). To exclude a compensatory contribution from other PGE_2_ synthases, we measured mPGES‐1 and cPGES and found neither was affected by *Ptges2* knockout (Figure S5a, Appendix S1).

Next, we examined the expression of four prostaglandin E_2_ receptors (EP), the downstream targets of PGE_2_, and found that all were upregulated with aging (Figure S5b, Appendix S1). However, *Ptger1* (EP1) was the only receptor whose expression was significantly downregulated by *Ptges2* knockout in vivo (Figure 5d), and the only receptor selectively induced by D‐galactose in MPC5 podocytes (Figure S5c, Appendix S1), suggesting that EP1 may be the preferentially inducible PGE_2_ receptor in podocytes under senescence‐inducing conditions.

Functionally, exogenous PGE_2_ reduced podocyte viability and aggravated cell senescence in MPC5 cells (Figure S5d–g, Appendix S1), supporting a pro‐senescent role of PGE_2_ in podocytes. To assess whether this response is cell‐type biased within the kidney, we performed parallel experiments in HK‐2 tubular epithelial cells. PGE_2_ exposure in HK‐2 tubular epithelial cells induced early changes in p16, p21, and p53 expression and partially reduced cell viability, but did not produce a robust SA‐β‐gal‐positive senescence phenotype within the experimental time frame (Figure S5h–l, Appendix S1). This cell type‐biased susceptibility, consistent with the limited regenerative capacity and intrinsic vulnerability of terminally differentiated podocytes, reinforces why podocyte mPGES‐2 is the rate limiting intrarenal node of the aged kidney.

Based on these observations, we further tested the involvement of the PGE_2_/EP1 axis by genetic and pharmacological approaches. *Ptges2* knockdown increased cell viability and attenuated podocyte senescence, whereas these protective effects were reversed by exogenous PGE_2_ administration (Figure S6a–e, Appendix S1). In contrast, *Ptges2* overexpression inhibited cell growth and accelerated cell senescence, both of which were partially blocked by SC51089, an EP1 receptor antagonist (Figure S6f–j, Appendix S1). Collectively, these findings support the notion that mPGES‐2 promotes podocyte senescence and accelerates renal aging, at least in part, through the PGE_2_/EP1 signaling pathway.

### 2.7 Podocyte mPGES‐2 Deficiency Protects Against Osteoporosis via the Kidney–Bone Axis

As shown in Figure S1a, *Ptges2* knockout improved bone architecture in aged mice, prompting us to investigate the role of mPGES‐2 in age‐related bone loss. Because osteoporosis frequently coexists with renal aging and chronic kidney injury (Caldiroli et al. 2025), we considered the possibility that mPGES‐2 might influence skeletal aging in parallel with its renal effects. Micro‐computed tomography showed that aging‐induced osteoporosis was markedly ameliorated in *Ptges2*‐knockout mice, as reflected by improved trabecular microarchitecture and increased bone mineral density (BMD; Figure 5e,f).

Bone homeostasis is maintained by the balance between osteoblast‐mediated bone formation and osteoclast‐mediated bone resorption (Zhu et al. 2020). Consistent with improved skeletal integrity, *Ptges2* knockout reduced cartilage degeneration and favored osteoblast formation, as shown by safranin O‐fast green, toluidine blue, tartrate‐resistant acid phosphatase (TRAP), and alkaline phosphatase (ALP) staining (Figure S7a–d, Appendix S1). Accordingly, osteoblast‐associated markers increased, whereas osteoclast‐associated markers decreased in aged *Ptges2*‐knockout mice (Figure 5g). These data indicate that loss of mPGES‐2 preserves bone homeostasis during aging.

**FIGURE 6:**
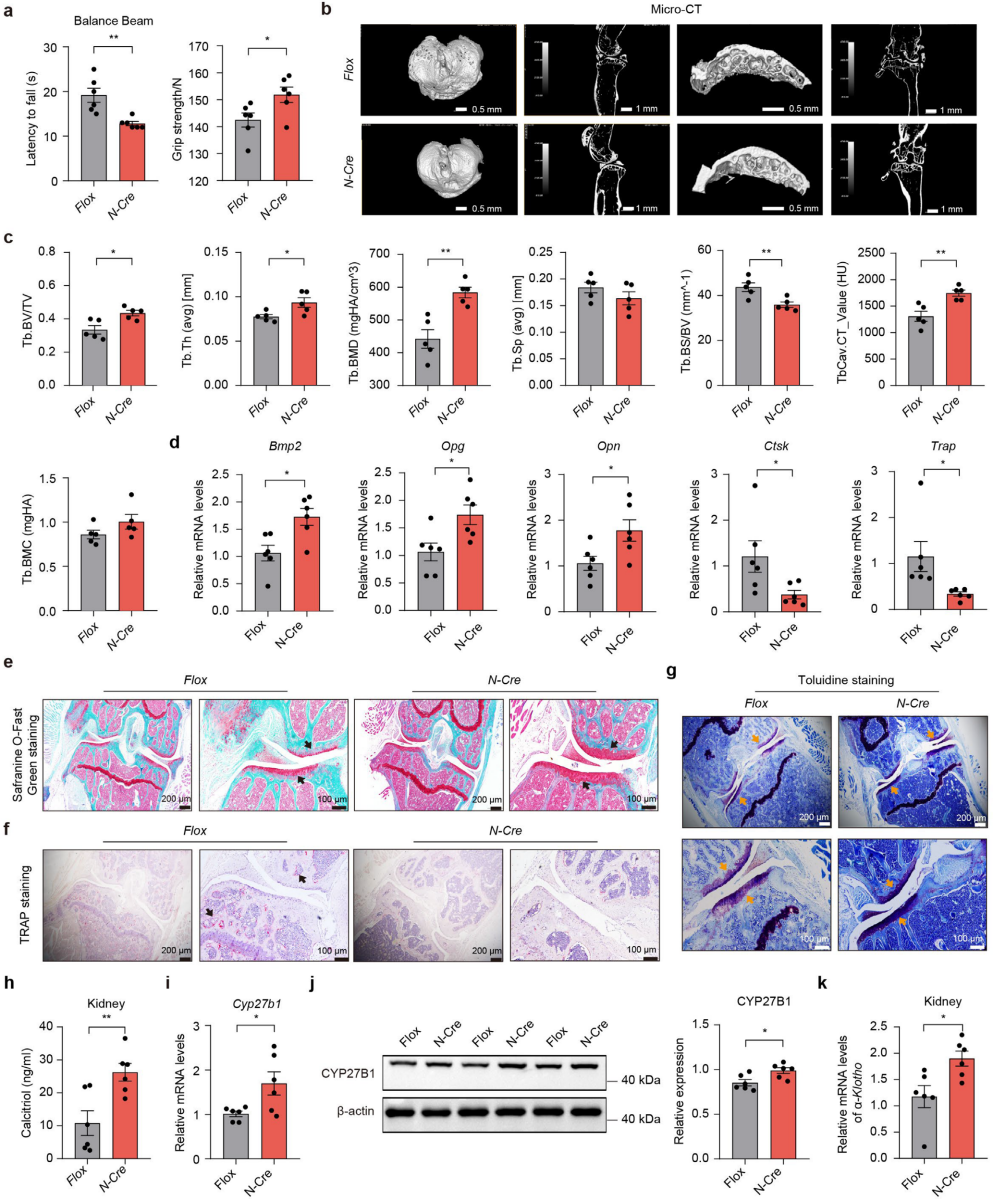
Effect of podocyte‐specific *Ptges2* knockout on bone formation and health. (a) Effect of podocyte‐specific *Ptges2* knockout on motor activity. (b) Bone microarchitecture by micro‐computed tomography (μCT). (c) Quantified indices by μCT. (d) Markers of osteogenesis and osteoclast resorption of podocyte *Ptges2* specific knockout mice. (e) Cartilage damage evaluated by Safranin O‐Fast Green Staining. (f) Osteoclastic bone resorption by tartrate‐resistant acid phosphatase (TRAP) staining. (g) Bone structural and cellular features assessment by toluidine blue staining of podocyte specific *Ptges2* knockout mice. (h) Calcitriol content in the kidney. (i) *Cyp27b1* mRNA levels in the kidney. (j) Representative blots and quantification of CYP27B1 in the kidney. (k) The mRNA levels of *α‐klotho* in the kidney. Data are presented as mean ± SEM. *p < 0.05, **p < 0.01, and ***p < 0.001; unpaired Student's *t*‐test was used for statistical analysis.

We next asked whether podocyte‐specific *Ptges2* deletion exerts a similar skeletal effect. Indeed, mice lacking *Ptges2* in podocytes showed improved motor activity and grip strength (Figure 6a). In parallel, osteoporosis and imbalanced mineral metabolism were substantially alleviated in podocyte‐specific *Ptges2*‐knockout mice (Figure 6b–d), as observed in global *Ptges2* knockout mice. Cartilage degeneration was reduced, osteoclast‐related markers declined, and osteoblast‐related markers increased (Figure 6e–g). Thus, podocyte mPGES‐2 contributes not only to renal aging but also to age‐related deterioration of bone.

To determine whether this skeletal phenotype reflected a direct action of PGE_2_ within bone, we measured bone PGE_2_ content and found no significant change following *Ptges2* knockout (Figure S8a, Appendix S1), arguing against a dominant bone autonomous PGE_2_ mechanism. Given the established kidney‐bone axis (Massy and Drueke 2020; Quarles 2008), we next examined renal endocrine mediators relevant to bone health. Calcitriol [1,25(OH)_2_D₃] is the active form of vitamin D and is normally produced in the kidneys (Selamet et al. 2018; Yoon et al. 2023), where adequate levels are necessary for proper bone mineralization and for balanced bone remodeling through regulation of osteoblast and osteoclast activity (Parfitt et al. 1984; Tang et al. 2019). Calcitriol can also influence α‐klotho, a kidney derived hormone tightly linked to systemic aging (Kurosu et al. 2005; Lau et al. 2012; Xu and Sun 2015).

In the present study, we found that *Ptges2* knockout increased calcitriol levels in aged mice (Figure S8b, Appendix S1). Since calcitriol is produced by CYP27B1 in the kidneys, the key activating enzyme involved in its synthesis, we observed the mRNA levels of *Cyp27b1* and found that it was upregulated in *Ptges2* knockout mice (Figure S8c, Appendix S1). Renal and circulating α‐klotho levels were likewise increased in aged *Ptges2*‐knockout mice (Figure S8d, Appendix S1). Importantly, podocyte‐specific *Ptges2* deletion also enhanced calcitriol production, accompanied by increased CYP27B1 expression (Figure 6h–j), and increased renal α‐klotho abundance (Figure 6k). These data suggest that podocyte mPGES‐2 influences renal endocrine function relevant to bone homeostasis.

To investigate the underlying mechanism, we collected conditioned medium from D galactose treated *Ptges2* overexpressing MPC5 podocytes (OE) and applied it to renal tubular cells. Conditioned medium from OE podocytes contained significantly higher PGE_2_ than control medium (Figure S9a, Appendix S1) and reduced CYP27B1 and α‐klotho expression in HK2 cells at both mRNA and protein levels (Figure S9b,c, Appendix S1), supporting a paracrine glomerulotubular interaction in which podocyte derived PGE_2_ downregulates the tubular endocrine program. Together, these findings support an indirect kidney to bone mechanism in which elevated podocyte mPGES‐2 contributes to age related skeletal decline by impairing the renal endocrine support required for bone homeostasis.

### 2.8 Pharmacological Inhibition of mPGES‐2 With SZ0232 Attenuates Renal Aging and Improves Bone Health

**FIGURE 7:**
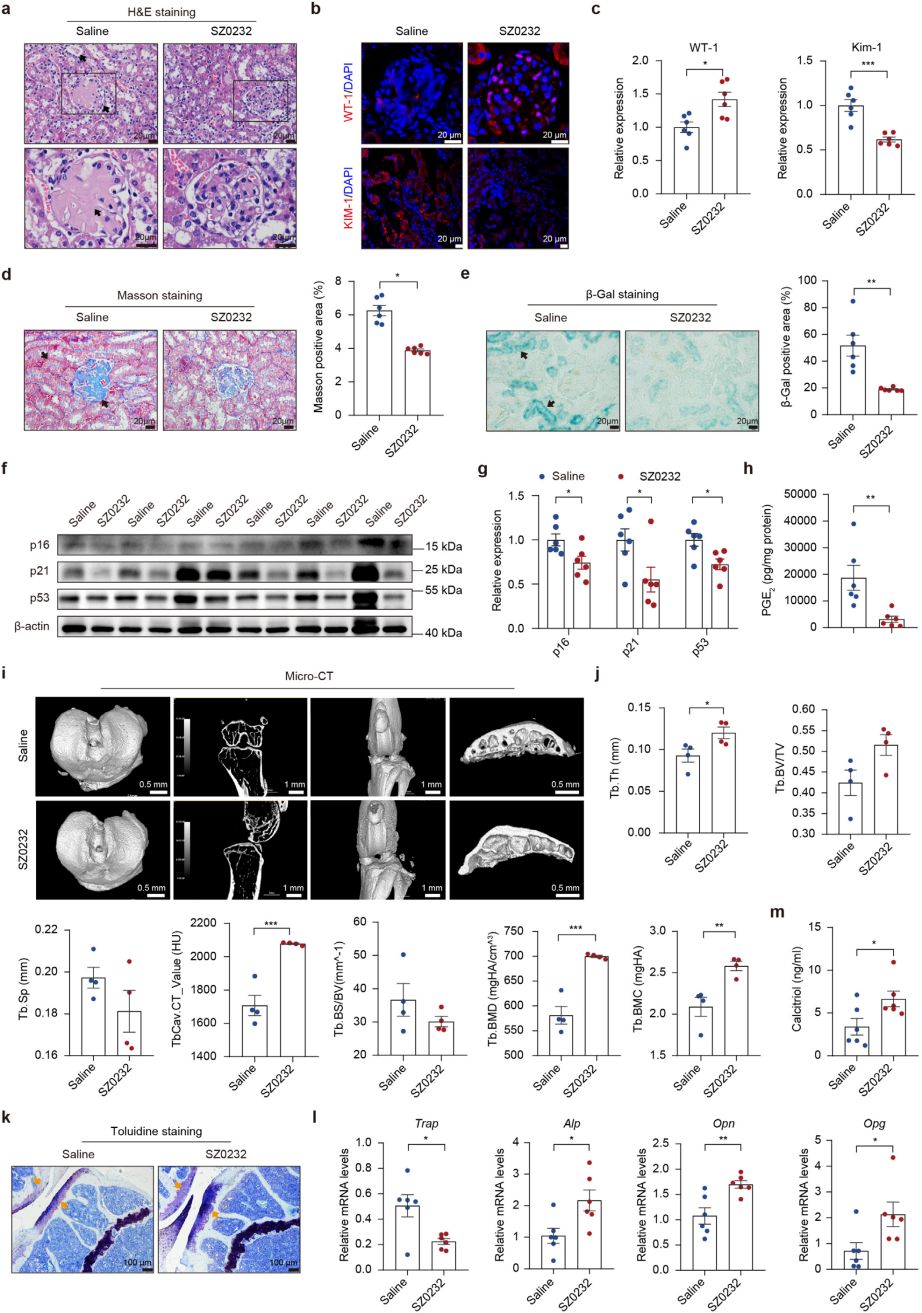
mPGES‐2 inhibitor SZ0232 alleviates aging‐related kidney dysfunction and promotes bone health. (a) Representative H&E staining of kidney tissues. (b) Representative images of WT‐1 and KIM‐1 by immunofluorescence. (c) Quantification of WT‐1 and KIM‐1. (d) Renal fibrosis analyzed by Masson's trichrome staining. (e) Renal aging analyzed by β‐Gal staining. (f) Representative blots of p16, p21, and p53 in the kidneys. (g) Quantification of p16, p21, and p53 in the kidney. (h) The content of PGE_2_ in the kidney. (i) Bone microarchitecture by μCT. (j) Quantification of indices by μCT. (k) Bone structural and cellular features assessed by toluidine blue staining. (l) mRNA levels of biomarkers of osteoblasts and osteoclasts. (m) Calcitriol content in the kidneys. Data are presented as mean ± SEM. *p < 0.05, **p < 0.01, and ***p < 0.001; unpaired Student's *t*‐test was used for statistical analysis.

To assess the translational potential of targeting mPGES‐2 in late life, we treated 20‐month‐old mice with SZ0232, a selective mPGES‐2 inhibitor identified by our group (Zhong et al. 2022), at 0.5 mg/kg intraperitoneally every 2 days for 2 months. SZ0232 improved aging‐associated injury in several organs, with the most prominent protection observed in the kidney (Figure 7a; Figure S10, Appendix S1), closely paralleling the phenotype of *Ptges2*‐knockout mice. SZ0232 reduced glomerulosclerosis, improved podocyte integrity, diminished renal fibrosis, and suppressed tubular apoptosis (Figure 7b–d; Figure S11a–d, Appendix S1). It also attenuated renal senescence, as shown by reduced SA‐β‐gal staining and lower expression of aging‐related proteins (Figure 7e–g). Consistent with the genetic models, SZ0232 lowered renal PGE_2_ levels (Figure 7h). In bone, SZ0232 improved trabecular microarchitecture, increased bone mineral density and bone mineral content, and rebalanced bone remodeling (Figure 7i–l; Figure S11e,f, Appendix S1), changes that were accompanied by increased renal calcitriol content (Figure 7m). These results support mPGES‐2 inhibition as a potentially actionable strategy for the treatment of renal aging and its skeletal complication.

## 3 Discussion

Here we identify mPGES‐2 as a previously unrecognized driver of renal aging and an important contributor to age‐related bone loss. Renal mPGES‐2 expression rose with age in both human and mouse kidneys, and either global or podocyte‐specific *Ptges2* deletion substantially attenuated renal injury and senescence in aged mice. The strong phenocopy between podocyte‐specific and global knockout models indicates that podocyte mPGES‐2 is a major intrarenal determinant of the aging kidney phenotype. Mechanistically, mPGES‐2 promoted podocyte senescence through a PGE_2_/EP1 program and concurrently disrupted renal endocrine outputs that support bone homeostasis. Pharmacological inhibition with SZ0232 reproduced the renal and skeletal benefits observed in the genetic models. Together, these data establish podocyte mPGES‐2 as a mechanistically informative and potentially druggable node in renal aging to its skeletal complication.

Despite a heavy clinical burden, renal aging still lacks a coherent molecular framework that links cell intrinsic injury within the kidney to systemic consequences in other organs. Aging‐associated structural changes, including nephron loss, glomerulosclerosis, and tubulointerstitial fibrosis, impair filtration, electrolyte balance, and renal hormone production, collectively predisposing to systemic complications such as bone disease (Boskey and Coleman 2010; Hommos et al. 2017; Luyckx et al. 2022; Razzaque 2007; Shen et al. 2024). By identifying a podocyte‐centered, mPGES‐2‐dependent program that simultaneously drives glomerular aging and renal endocrine dysfunction, our work begins to fill this gap and offers a unifying explanation for the frequent coexistence of CKD and senile osteoporosis in older populations.

Although identified over two decades ago, mPGES‐2 has received far less attention than mPGES‐1, and its biology has remained somewhat paradoxical since the recognition that it can generate MDA rather than PGE_2_ under many in vivo conditions (Lindner et al. 2007; Nitz et al. 2007). Consistent with this view, our previous studies and others have shown that *Ptges2* manipulation does not measurably alter renal PGE_2_ levels in diabetic kidney disease or acute kidney injury (Zhong, Cai, et al. 2023; Zhong et al. 2024, 2022). The present work resolves this apparent paradox by revealing an unexpected context dependence: during natural aging, *Ptges2* deletion lowers renal PGE_2_ without altering MDA, indicating that mPGES‐2 engages a PGE_2_‐relevant program in the aged kidney. Single‐cell transcriptomic analysis localized *Ptges2* preferentially to podocytes among glomerular cell types, and podocyte‐specific deletion nearly recapitulated the renoprotective effect of global knockout. These observations place terminally differentiated podocytes at the center of the mPGES‐2 dependent aging program and suggest that the dual function nature of mPGES‐2 is reinterpreted in a cell type and context specific manner.

Mechanistically, our data support a model in which mPGES‐2 drives podocyte senescence through PGE_2_/EP1 signaling. Among the four EP receptors, EP1 was the only receptor whose expression was downregulated by *Ptges2* knockout in vivo and selectively induced by D galactose in cultured podocytes. Exogenous PGE_2_ aggravated senescence in MPC5 cells, *Ptges2* knockdown was rescued by PGE_2_, and the phenotype caused by *Ptges2* overexpression was blocked by the EP1 antagonist SC51089. Although these findings do not exclude additional downstream mediators, they strongly implicate EP1 as the dominant receptor through which mPGES‐2 linked PGE_2_ signaling promotes podocyte aging. In parallel experiments using HK‐2 tubular epithelial cells, PGE_2_ elevated p16, p21, and p53 transcripts and reduced viability but failed to induce a robust SA β galactosidase positive phenotype within the time frame tested, indicating that the senescence response of tubular cells is comparatively attenuated. This cell type biased susceptibility is consistent with the limited regenerative capacity and intrinsic vulnerability of terminally differentiated podocytes and reinforces why podocyte mPGES‐2 is the rate limiting node of the aging kidney.

Several lines of evidence support the human relevance of these findings. Our human tissue array showed a positive correlation between renal mPGES‐2 expression and donor age, and genetic ablation in mice suppressed renal PGE_2_ synthesis, confirming a direct biochemical link consistent with the cortex enriched constitutive expression of mPGES‐2 (Nasrallah et al. 2016). At the population level, the Rotterdam Study (*n* = 2291, age ≥ 55) showed that urinary PGE_2_ rose with age, was independently associated with lower eGFR, and prospectively predicted incident eGFR < 60 mL/min/1.73 m^2^ (HR 1.13, 95% CI 1.02–1.25) (Geurts et al. 2024), echoing earlier work linking PGE_2_ receptor signaling to renal injury and fibrosis (Nasrallah et al. 2014). Human genetics further implicate this pathway: variants in *PTGER1* (EP1) and *PTGER4* are associated with biopsy proven nephrosclerosis (Ware and Howard 1993). These data align with podocyte senescence as a central driver of renal aging (Li and Liu 2025; Schmitt and Melk 2017), and together support the translational relevance of targeting the mPGES‐2/PGE_2_/EP1 axis in renal aging.

A distinctive feature of this study is the explicit connection between renal aging and skeletal decline. The bone phenotype was improved not only in global knockout mice but also in podocyte‐specific knockout mice, arguing that the skeletal benefit is not merely an unrelated consequence of systemic gene deletion. Because bone PGE_2_ was unchanged, we favor an indirect kidney‐bone mechanism rather than a direct effect of mPGES‐2 on bone tissue. Both global and podocyte‐specific *Ptges2* deletion restored renal calcitriol and α‐klotho, two endocrine outputs with well‐established roles in mineral metabolism and bone remodeling (Agoro and White 2023; Komaba et al. 2017). Critically, conditioned medium from *Ptges2*‐overexpressing podocytes exhibited evaluated PGE_2_ levels and suppressed CYP27B1 and α‐klotho expression in tubular cells, providing direct evidence for paracrine glomerulotubular communication. Calcitriol and α‐klotho are important, but not necessarily exclusive, mediators of this axis; additional podocyte derived or kidney derived factors may also contribute and remain to be defined. We therefore propose that podocyte mPGES‐2 promotes age related osteoporosis, at least in part, by impairing the renal endocrine support required for bone homeostasis.

Several limitations should be acknowledged. First, although the global knockout cohort allowed lifespan analysis and broad safety assessment, analogous lifespan studies were not performed in the conditional knockout cohorts or in SZ0232 treated mice; whether podocyte specific *Ptges2* deletion or pharmacological inhibition extends lifespan therefore remains unresolved. Second, the age dependent increase in *Ptges2* within podocytes is primarily supported by single cell transcriptomic analysis, and reliable spatial analysis in aged mouse glomeruli was further limited by severe glomerulosclerosis and distortion of glomerular architecture. Third, the human kidney specimens used in this study were obtained from a commercial tissue array without matched clinical renal function metadata (eGFR or serum creatinine), and histologically normal fresh kidney tissue from healthy aged donors was not available; consequently, neither colocalization nor a direct correlation between mPGES‐2 expression and renal functional decline could be established in this cohort. Fourth, our loss and gain of function experiments were performed in MPC5 podocytes, and whether the same mPGES‐2/PGE_2_/EP1 senescence program operates in primary aged podocytes, other renal cell types, or extra renal tissues remains to be defined. Finally, although calcitriol and α‐klotho were identified as key endocrine outputs linking renal aging to skeletal decline, additional podocyte derived or kidney derived mediators downstream of mPGES‐2 may also contribute to the kidney to bone axis and to renal aging more broadly. These issues warrant further study.

In summary, our findings identify mPGES‐2 as a key determinant of renal aging and a contributor to age‐related osteoporosis. By integrating human tissue analysis, single‐cell transcriptomics, conditional genetics, and pharmacological intervention, we show that podocyte mPGES‐2 drives aging‐associated kidney injury and influences skeletal integrity through a renal endocrine mechanism. The protective effects of SZ0232 further support the translational potential of targeting mPGES‐2 in late life. These results broaden the current framework of renal aging and nominate mPGES‐2 as a therapeutic target for aging‐associated kidney and bone disorders.

## 4 Materials and Methods

### 4.1 Reagents

The sources of key reagents and materials are detailed in Table S1 (Appendix S1).

### 4.2 Mice

All animals were maintained in a specific pathogen‐free animal facility at an ambient temperature of 22°C and 50% humidity under a 12/12 h light/dark cycle, with free access to water and food. All experimental protocols were performed in strict accordance with the recommendations of the Guide for the Care and Use of Laboratory Animals of the China Association for Laboratory Animal Science and were approved by the Animal Ethics Committee of Xuzhou Medical University (202301T006). C57BL/6J *Ptges2* ^*+/−*^ and *Ptges2* ^*flox/+*^ mice were generated by GemPharmatech Co. Ltd. (Nanjing, China) using CRISPR/Cas9 technology. Nphs‐2 Cre mice were gifted by Professor Zhanjun Jia of Nanjing Medical University. *Ksp‐Cre* transgenic mice were gifted by Professor Baoxue Yang of Peking University. *Ptges2* ^*−/−*^ mice were obtained by self‐crossing C57BL/6J *Ptges2* ^*+/−*^ mice. Podocyte‐specific *Ptges2* knockout mice (*Ptges2* ^*f/f*^ *;Nphs2‐Cre*) were generated by crossbreeding *Nphs‐2 Cre* mice with *Ptges2* ^*flox*/+^ mice. Tubule‐specific *Ptges2* knockout mice (*Ptges2* ^*f/f*^ *;Ksp‐Cre*) were generated by crossbreeding *Ksp‐Cre* transgenic mice with *Ptges2* ^*flox*/+^ mice. Littermate *Ptges2* ^*f/f*^ mice served as controls. *Ptges2* knockout was confirmed by tail genotyping and western blotting as previously reported (Zhong et al. 2024).

### 4.3 mPGES‐2‐Inhibitor Treatment

Here, 20‐month‐old mice fed a standard laboratory chow diet were administered either SZ0232 or saline control. SZ0232 was injected intraperitoneally at a dose of 0.5 mg/kg once every 2 days for 2 months. SZ0232 was dissolved in a saline solution at 0.1 mg/mL. At the end of the experimental period, blood and urine samples were collected, and the mice were sacrificed to harvest the kidney, liver, brain, heart, muscle, bone, and fat tissues for further analysis.

### 4.4 Cell Lines

HEK293T cells were purchased from ATCC and cultured in DMEM (C11995500BT, Gibco) supplemented with 10% heat‐inactivated fetal bovine serum (FBS) (A5256701, Thermo Fisher Scientific) and 1X penicillin–streptomycin solution (C0222, Beyotime Biotechnology). The conditionally immortalized murine podocyte cell line (MPC5) was purchased from BNCC (Beina Biotechnology Co. Ltd.). MPC5 cells were cultured in RPMI 1640 medium (C11875500BT, Thermo Fisher Scientific) supplemented with 10% FBS and 100 U/mL penicillin plus 0.1 mg/mL streptomycin. All cells were grown at 37°C in a humid atmosphere containing 5% CO_2_.

### 4.5 Cell Treatment

D‐galactose was used to induce cell senescence. Cells were exposed to D‐galactose (G0750‐25G, Sigma‐Aldrich) at a dose of 15 mg/mL for 60 h. 16,16‐dimethyl Prostaglandin E_2_ (14,750, Cayman Chemical) was added to cells for 36 h. SC51089 (HY‐108563, MedChemExpress) was added to the medium for 60 h at a final concentration of 10 μM. After the experiments, cells were collected for further analysis. In addition, MPC5 podocytes stably overexpressing *Ptges2* (OE) or carrying the empty vector (NC) were cultured overnight and switched to serum free medium containing D‐galactose (15 mg/mL) for 24 h. Conditioned medium was clarified by centrifugation, and PGE_2_ concentration was measured by ELISA (Cayman 514010). HK2 cells at 70%–80% confluence were then incubated with NC‐CM or OE‐CM (1:1 with fresh DMEM) for 48 h. *Cyp27b1* and *α‐klotho* mRNA were quantified by qRT‐PCR; CYP27B1 and α‐klotho protein by western blotting.

### 4.6 Lentivirus‐Mediated Gene Expression and Interference

The mouse *Ptges2* gene was cloned into the expression vector pCDH‐CMV2‐puro (Jiangsu Laisen Co. Ltd.). The shRNA sequence targeting mouse *Ptges2* (5′‐CGTGAGAAGGACTGAGATCAA‐3′) was designed and inserted into the lentiviral expression vector pLKO.1‐puro (Shanghai GenePharma Co. Ltd.). To produce lentiviral particles, HEK293T cells were transiently transfected with the recombinant plasmids along with the packaging plasmids psPAX2 and pMD2.G for 48 h. The viral supernatant was then collected, filtered, and used to infect MPC5 cells for 48 h. Infected cells were subsequently selected with puromycin dihydrochloride (ab141453; Abcam) for an additional 72 h.

### 4.7 Analysis of Human Kidney Sections

Chips of human kidney tissues were provided by ZK Biotechnology Co. Ltd. The organ donors are men and women aged from 16 to 82 year‐old with informed consent. In all cases, informed written consent was collected by the company where the organs were recruited. For this human experiment, the study was approved by the Human Ethics Committee of ZK Biotechnology Co. Ltd. The detailed information of donors was listed in Table S2 in Appendix S1.

### 4.8 Physical Function Tests

Motor coordination and capability were evaluated using a balance‐beam task. Prior to beginning the experiment, mice were acclimated to the testing area for at least 60 min, starting from a wider beam to the narrow testing beam, in which mice were placed at the start and allowed to traverse the beam to reach the endpoint, repeating four consecutive trials. After training, the mice were allowed to walk across a narrow beam, and the event of falling from the beam was recorded. The test was performed 3 times and the overall score for each mouse was the average of the three trial scores. Forelimb grip strength (N‐F/kg) is determined using a Grip Strength Meter (Columbus Instruments, Columbus, OH). The mice were placed on the grid, keeping the torso horizontal, and allowing only their forepaws to attach to the grid before measurements were taken. The mouse was gently pulled back by its tail, ensuring that the mouse gripped the top portion of the grid and the torso remained horizontal, and the maximal grip strength value of the mouse was recorded and displayed on the screen. This procedure was performed 10 times and the mean value was calculated to obtain forelimb grip strength measurements.

### 4.9 Histological Analysis

Multiple tissues isolated from the above mouse models were fixed in 4% paraformaldehyde overnight. Tissues were embedded in paraffin and cross‐sectioned for histological analysis. As for Hematoxylin–eosin (H&E) and PAS staining, the paraffin‐embedded sections were de‐paraffinized, dehydrated with xylene and ethanol, and successively stained with H&E or PAS (Crowley et al. 2009; Fu et al. 2020). Masson's trichrome staining was performed using a kit (G1340, Solarbio), according to the manufacturer's instructions. Sirius red staining was conducted by Servicebio Technology Co. Ltd. As for immunohistochemical analysis, the prepared slides were stained with the indicated primary antibodies. As for transmission electron microscopy (TEM) analysis, fresh kidney cortex samples were collected and immediately fixed in 2.5% glutaraldehyde. The samples were then performed for TEM by Servicebio Technology Co. Ltd. Images were systematically captured using an Olympus BX43F microscope.

### 4.10 Osteoblasts‐ and Osteoclasts‐Specific Staining

Alkaline phosphatase (ALP) and tartrate‐resistant acid phosphatase (TRAP) are marker enzymes for osteoblasts and osteoclasts, respectively, and these enzymes are markers that highlight the presence of osteoblasts and osteoclasts (Sun et al. 2020; Zeng et al. 2016). Briefly, bone tissue was fixed with 4% paraformaldehyde, decalcified, and embedded in paraffin. Paraffin sections were prepared and deparaffinized for staining with ALP (D001‐2‐2, Nanjing Jiancheng Bioengineering Institute) and TRAP according to the instructions of the indicated commercial kits (G1050‐50T, Servicebio Technology Co. Ltd.). Images were captured using an Olympus BX43F microscope.

### 4.11 Cartilage‐Specific Matrix Staining

Safranin O‐Fast Green, and toluidine blue staining were used to detect cartilage, mucin, and mast cell granules in the bone (Hu and Olsen 2016). For Safranin O‐Fast Green staining (G1371, Solarbio), deparaffinized sections were stained with Weigert's iron hematoxylin working solution for 5 min and washed gently in distilled water for 10 min. Sections were stained with a fast green solution for 5 min and rinsed quickly with a 1% acetic acid solution for 10–15 s. Images were captured using an Olympus BX43F microscope. For toluidine blue staining (G2543, Solarbio), deparaffinized sections were stained with a toluidine blue working solution for 2–3 min and rinsed gently with three changes of distilled water for 3 min. Next, the slides were dehydrated with 95% absolute alcohol, followed by two changes of xylene and coverslips.

### 4.12 Micro‐Computed Tomography (μCT) Assays

Micro‐computed tomography (μCT) was used to evaluate bone microstructure and mineralization. The femurs were collected, and soft tissues were removed. The femurs were then placed with gauze in a sample holder and scanned using CT (VNC‐102) at the Shanghai Aiyou Biotechnology Center. The images were captured using Cruiser and analyzed using Avatar software. Trabecular morphometry was characterized by measuring bone mineral density (BMD), bone volume fraction (BVF), bone surface area to bone volume ratio (BS/BV), bone volume fraction (BV/TV; %), trabecular thickness (Tb.Th), trabecular number (Tb.N), and trabecular spacing/separation (Tb.Sp). All μCT parameters were derived using the manufacturer's protocols.

### 4.13 Immunofluorescence Staining

Frozen tissue sections were prepared for immunofluorescence staining. Frozen sections were thawed and washed with PBS. After that, the sections were incubated with 0.3% Triton X‐100 for 1 h and blocked with 10% goat serum for 1 h at room temperature. The sections were then incubated with the indicated primary antibody for 12 h at 4°C. The primary antibodies used were as follows: Wilms tumor protein (ab89901, Abcam), nephrin (AF3159, R&D systems), synaptopodin (sc‐515842, Santa Cruz), *lotus tetragonolobus* lectin (L32480, Thermo Fisher Scientific), and mPGES‐2 (10881‐1‐AP, proteintech). Detection was performed using the following secondary antibodies: Donkey Anti‐Mouse IgG (H+L) Dylight488 (E032211, Earthox), Goat Anti‐Rabbit IgG (H+L) Dylight488 (E032220, Earthox), Goat Anti‐Mouse IgG (H+L) Dylight594 (E032410, Earthox), and Goat Anti‐Rabbit IgG (H+L) Dylight594 (E032420, Earthox). Nuclei were counterstained with DAPI (C1002, Beyotime Biotechnology). Confocal images were captured using a Leica STELLARIS 5 microscope with Leica Application Suite X (LAS X) imaging software. For image quantification, podocyte markers were calculated specifically in glomerular areas. Images were quantified using the Image J software. The detailed information of donors was listed in Table S2 in Appendix S1.

### 4.14 β‐Gal Staining

Frozen tissue sections were prepared for β‐Gal staining according to the manufacturer's instructions (G1580, Solarbio). Briefly, the prepared slides were fixed and incubated with the SA‐β‐Gal staining solution in a humidified chamber and protected from light at 37°C for 4 h to overnight. When the color reaction was complete, rinsing in 1X PBS and mounted with coverslips. Images were quantified using the ImageJ software. β‐Gal positive areas were quantified using the Image J software.

### 4.15 TUNEL Assays

Terminal deoxynucleotidyl transferase dUTP nick end labeling (TUNEL) assay relies on the enzyme terminal deoxynucleotide transferase (TdT), which attaches deoxynucleotides to the 3′‐hydroxyl terminus of DNA breaks. This assay is widely used to detect apoptosis and cell death. For the TUNEL assay, frozen tissues were fixed with 4% paraformaldehyde for 4–24 h at 4°C. After fixation, the tissues were cut into thin slices of 10 μm or less. The prepared slices were then permeabilized, which allowed reagents, such as the TdT enzyme, to penetrate the cell nucleus according to a commercial kit (MA0223‐1, MeilunBio). To quantify apoptotic cells, the number of TUNEL‐positive (green) cells was counted and divided by the total number of cells in each field using the ImageJ software.

### 4.16 Serum BUN and Creatinine Measurement

Blood samples were collected from the orbital vein and centrifuged to collect serum. Serum blood urea nitrogen (BUN) and creatinine levels were measured using commercial kits (C013‐2‐1, Nanjing Jiancheng Bioengineering Institute; C011‐2‐1, Nanjing Jiancheng Bioengineering Institute) according to the manufacturer's instructions.

### 4.17 α‐Klotho Measurement

Serum and kidney samples were prepared, and the content of α‐Klotho was detected according to the procedure of a commercial kit (SEH757Mu, Cloud‐Clone Corp.).

### 4.18 Glomerular Filtration Rate (GFR) Measurement

GFR was measured transcutaneously using a MediBeacon transdermal GFR monitor (MediBeacon Inc., Mannheim, Germany). Briefly, the flank region of each mouse was depilated one day prior to measurement. On the day of the experiment, mice were anesthetized with isoflurane and the sensor was affixed to the depilated skin. A 5‐min baseline signal was acquired before a single bolus of FITC‐sinistrin (150 mg/kg body weight) was administered via tail vein injection. Mice were then individually housed and transcutaneous fluorescence was recorded continuously for 90 min. GFR values (μL/min per 100 g body weight) were calculated from the fluorescence decay curve using MPD Studio software (MediBeacon Inc.).

### 4.19 Measurement of Urinary Albumin Levels

Urine samples were collected using metabolic cages in which mice were provided with food and water ad libitum. The collected urine samples were centrifuged to remove debris. The supernatant protein and creatinine concentrations in the urine samples were measured using a Mouse Urine Albumin Assay Kit (EIA06044m; Wuhan Xinqidi Biotech Co. Ltd.) and a Urine Creatinine Kit (C011‐2‐1; Nanjing Jiancheng Bioengineering Institute), respectively, according to the manufacturer's instructions. Urinary albumin levels are reported as the ratio of urinary albumin to creatinine.

### 4.20 PGE_2_ Measurement

Kidney cortical samples were homogenized in phosphate‐buffered saline (PBS) containing 1 mM ethylenediaminetetraacetic acid and 10 μM indomethacin. Samples were centrifuged at 10,000 × g for 15 min at 4°C to collect supernatants. The levels of PGE_2_ in the supernatants were measured using an ELISA kit (514010, Cayman Chemical) according to the manufacturer's instructions.

### 4.21 MDA Measurement

Kidney cortical samples were homogenized on ice in 300 μL of the malondialdehyde (MDA) lysis buffer containing BHT. The samples were centrifuged at 13,000 × *g* for 10 min to remove insoluble material. Thereafter, 200 μL of the supernatant from each homogenized sample was placed in a microcentrifuge tube. The MDA concentration was measured using an MDA assay kit (A003‐4‐1, Nanjing Jiancheng Bioengineering Institute) according to the manufacturer's instructions.

### 4.22 Kidney Calcitriol Measurement

Calcitriol1,25‐Dihydroxycholecalciferol (1,25‐dihydroxyvitamin D_3_) is the active form of vitamin D normally present in the kidneys. The detection of calcitriol is based on competitive enzyme‐linked immunosorbent assay technology, in which TMB is converted into blue under the catalysis of peroxidase and then converted into the final yellow color under the action of acid. The intensity of the yellow color was inversely proportional to the amount of calcitriol bound to the plate. Briefly, the kidney tissue was homogenized in PBS to obtain the supernatant. The kit components and samples were equilibrated at room temperature before use. Supernatants were processed and analyzed using a commercial kit (E‐HS30655Mo, Enovabio).

### 4.23 qRT‐PCR

RNA samples were isolated using TRIzol reagents (15596026CN, Thermo Fisher Scientific). cDNA was generated via reverse transcription using the HyperScript III RT SuperMix for qPCR with gDNA Remover (R202‐02, Enzyartisan). The specific primers for target genes were designed using the Primer3 software (available at http://frodo.wi.mit.edu/primer3/) and were listed in the Table S3 of Appendix S1. qRT‐PCR amplification was performed using 2 × S6 Universal SYBR qPCR Mix (Q204‐01, Enzyartisan). Levels of the housekeeping gene beta‐actin were used as an internal control for the normalization of RNA quantity and quality differences among the samples. The qRT‐PCR data were acquired using Roche LightCycler 480 II/96 with the Light Cycler 480 SW 1.5 software. The fold changes in gene expression were normalized to beta‐actin expression.

### 4.24 Western Blot Analysis

Tissues and cell pellets were collected and resuspended in RIPA lysis buffer and homogenized to lysates. The lysates were incubated on ice for 30 min and centrifuged at 4°C for 15 min at 12,000 g. Protein levels of soluble supernatant were estimated using a BCA Protein Assay Kit (23227, Thermo Fisher Scientific) according to the manufacturer's protocols. Equal amounts of proteins were applied to a 10 or 12.5% sodium dodecyl sulfate‐polyacrylamide gel for electrophoresis to separate proteins, after which the proteins were electrophoretically transferred onto nitrocellulose membranes (Millipore). The membranes were blocked using 3% bovine serum albumin (A8020, Solarbio) for 1 h at room temperature and then incubated with indicated antibodies overnight at 4°C. In this study, the following antibodies were used: p16 (SC‐1661, Santa Cruz), p21 (SC‐6246, Santa Cruz), p53 (SC‐126, Santa Cruz), mPGES‐2 (10881‐1‐AP, Proteintech). The secondary antibodies: HRP‐labeled Goat Anti‐Mouse IgG (H+L) (A0216, Beyotime) and HRP‐labeled Goat Anti‐Rabbit IgG (H+L) (A0208, Beyotime) were used. Protein bands were detected with an ECL Plus substrate (044–500 mL, Beijing Fluorescence Biotechnology Co. Ltd). Band intensities were analyzed using the ImageJ software.

### 4.25 Statistical Analysis

Statistical analyses were performed using GraphPad Prism version 8 (GraphPad Software Inc., La Jolla, CA, USA). All samples were randomly allocated to the treatment groups. All statistical measurements are presented as the mean ± standard error of the mean (SEM). Unpaired Student's *t*‐test (two‐tailed) was used to assess the statistical significance of an observed parameter between experimental groups. To compare more than two groups, data were analyzed using one‐way ANOVA, followed by Tukey's post hoc test. *p Values < 0.05 were considered statistically significant. Details regarding other materials and methods are provided in the Appendix S1.

## Author Contributions

Conceptualization: D.Z., Y.S., Z.J., J.C., and D.G. Methodology: D.Z., C.H., M.L., J.L., L.Z., S.N., Z.X., Yue. S., Y.Z., and D.S. Visualization: D.Z., C.H., M.L., and J.Z. Funding acquisition: D.Z., J.C., and Y.S. Supervision: D.Z., D.G., C.J., Z.J., and S.Y. Writing – original draft: D.Z. Writing – review and editing: D.Z., Z.J, H.G., J.C., and Y.S.

## Funding

This research was supported by the National Natural Science Foundation of China grant 82470744 (D.Z.), 82370681 (Y.S), 82293641 (J.C.); Key Project of Natural Science Research in Jiangsu Universities grant 21KJA350002 (Y.S.); Natural Science Foundation of Jiangsu Province grant BK20250158 (D.Z.), BK20240050 (Y.S.); Jiangsu Qinglan Project (D.Z.); Jiangsu Government Scholarship for Overseas Studies (D.Z.); Jiangsu Research Innovation Program for College Graduates grant KYCX24_3119 (L.L.Z.), KYCX25_3257 (S.N.), and Construction Project of High Level Hospital of Jiangsu Province grant LCZX202403 (Y.S.).

## Conflicts of Interest

The authors declare no conflicts of interest.

## Supporting information


**Figure S1:** Effect of mPGES‐2 deficiency on the morphological and functional changes of multiple tissues. (a) Morphological changes in different tissues assessed by (hematoxylin and eosin) H&E staining. (b) Serum ALT, AST, BUN, and creatinine levels. Data are mean ± SEM. One‐way ANOVA followed by Tukey's test was used for b. O‐KO, old‐knockout; O‐WT, old‐wildtype; Y‐KO, young‐knockout; Y‐WT, young‐wildtype.
**Figure S2:** Effect of mPGES‐2 deficiency on renal damage and podocyte dysfunction caused by aging. (a) Glomerular filtration rate (GFR). (b) The ratio of urine albumin to creatinine. (c) PAS staining of kidney tissues. (d) Quantification of mesangial matrix. (e) Cell death analyzed by TUNEL. (f) Quantification of TUNEL positive staining. (g) Renal fibrosis analyzed by Masson's trichrome staining. (h) Quantification of positive area of Masson's trichrome staining. (i) Renal fibrosis analyzed by Sirius red staining. (j) Quantification of Sirius red positive area. (k) Expression of senescence‐associated secretory phenotype (SASP) factors. Data are presented as means ± SEM. *p < 0.05, **p < 0.01, ***p < 0.001; One‐way ANOVA followed by Tukey's test was used for statistical analysis.
**Figure S3:** Podocyte *Ptges2* specific knockout alleviate renal dysfunction and fibrosis caused by aging. (a) Assessment of morphological changes by Periodic acid–Schiff (PAS) staining. (b) Renal fibrosis assayed by Sirius red staining. *N‐Cre*: *Ptges2* ^*f/f*^ *;Nphs2‐Cre*. *Flox*: *Ptges2* ^*f/f*^. Data are means ± SEM. *p < 0.05, **p < 0.01, ***p < 0.001; unpaired Student's *t*‐test was used for statistical analysis.
**Figure S4:** Tubule *Ptges2* specific knockout alleviates renal aging and associated kidney injury. (a) H&E staining. (b) Renal fibrosis assayed by Masson's trichrome staining. (c) Renal fibrosis assayed by Sirius red staining. (d) Cell death analyzed by TUNEL staining. (e) Quantification of TUNEL positive staining. (f) Cell senescence analyzed by β‐Gal staining. (g) mRNA levels of aging biomarkers (p16, p21 and p53). (h) Representative blots of aging biomarkers of p16, p21and p53. (i) Quantification of p16, p21 and p53 protein levels. (j) mRNA levels of SASP factors. *K‐Cre*: *Ptges2* ^*f/f*^ *;Ksp‐Cre*. *Flox*: *Ptges2* ^*f/f*^. Data are presented as means ± SEM. *p < 0.05, **p < 0.01; Unpaired Student's *t*‐test was used for statistical analysis.
**Figure S5:** PGE_2_ triggers podocyte and tubular cell senescence. (a) Effect of *Ptges2* knockout on the expression of three known PGE_2_ synthases. (b) Relative mRNA expression levels of *Ptger*1‐4 (EP1‐4) in kidneys from young and old wild‐type mice. (c) Relative mRNA expression levels of *Ptger*1‐4 in mouse podocyte cells (MPC5) from the control (NC) and D‐gal‐treated groups. (d) Effect of PGE_2_ on MPC5 cell viability. (e) Effect of PGE_2_ on MPC5 cell senescence assayed by β‐Gal staining. (f) Effect of PGE_2_ on mRNA levels of aging biomarkers in MPC5 cells. (g) Effect of PGE_2_ on mRNA levels of SASP factors in MPC5 cells. (h) Effect of PGE_2_ on human renal tubular epithelial cells (HK2) cell viability. (i) β‐Gal staining in HK‐2 cells. (j) Quantification of β‐Gal positive staining in HK‐2 cells. (k) mRNA levels of senescence markers in HK‐2 cells. (l) SASP factors in HK‐2 cells. Data are presented as means ± SEM. *p < 0.05, **p < 0.01, ***p < 0.001; Unpaired Student's *t*‐test was used for statistical analysis.
**Figure S6:** Effect of *Ptges2* knockdown and overexpression on podocyte cell senescence. (a–e) *Ptges2* knockdown and further exposed to PGE_2_ in MPC5 cells. (a) Cell viability. (b) Cell senescence assayed by β‐Gal staining. (c) Quantification of β‐Gal positive area. (d) mRNA levels of aging biomarkers. (e) mRNA levels of SASP factors. (f–j) The effect of *Ptges2* overexpression and further exposed to SC51089, an antagonist of EP1 receptor. (f) Cell viability. (g) Cell senescence assayed by β‐Gal staining. (h) Quantification of β‐Gal staining. (i) mRNA levels of aging biomarkers. (j) mRNA levels of SASP factors. Data are presented as means ± SEM. *p < 0.05, **p < 0.01, ***p < 0.001; ordinary one‐way analysis of variance (ANOVA) with Tukey's test was used for statistical analysis.
**Figure S7:** Effect of *Ptges2* knockout on bone structure and function. (a) Assessment of osteoclasts function by tartrate‐resistant acid phosphatase (TRAP) staining of *Ptges2* knockout and control mice. (b) Cartilage damage evaluated by Safranin O‐Fast Green Staining. (c) Bone structural and cellular features assessment by toluidine blue staining. (d) Assessment of osteoblasts function by ALP staining.
**Figure S8:** Effect of *Ptges2* knockout on hormones associated with bone health. (a) *Ptges2* knockout on the content of PGE_2_ in the bones. (b) *Ptges2* knockout on the content of calcitriol, also named as 1,25(OH)_2_D_3_, in the kidneys. (c) *Ptges2* knockout on the mRNA levels of *Cyp27b1* in the kidneys. (d) *Ptges2* knockout on the levels of α‐klotho in the kidney and serum. Data are presented as mean ± SEM. *p < 0.05 and **p < 0.01; unpaired Student's *t* test was used for statistical analysis.
**Figure S9:** Conditioned medium derived from *Ptges2*‐overexpressing podocytes suppressed tubular endocrine synthesis capacity. MPC5 podocytes were transfected with a *Ptges2* overexpression plasmid (OE) or the corresponding empty vector (NC). After 24 h, conditioned medium was collected and used to treat HK2 for 48 h. (a) PGE_2_ levels in conditioned medium collected from MPC5 cells. (b) Relative mRNA levels of *Cyp27b1* and *α‐klotho* in HK2 cells. (c) Representative western blots and densitometric quantification of CYP27B1 and α‐klotho protein levels in HK2 cells. Data are presented as mean ± SEM Statistical significance was assessed using an unpaired two‐tailed Student's *t*‐test. *p < 0.05, **p < 0.01.
**Figure S10:** Effect of SZ0232 on the morphological changes of multiple tissues at old age. Morphological changes in different tissues from mice treated with SZ0232 or saline assessed by (hematoxylin and eosin) H&E staining.
**Figure S11:** mPGES‐2 inhibitor SZ0232 protects against aging associated renal and bone dysfunction. (a) PAS staining of kidney tissues. (b) Immunofluorescence (IF) staining of podocyte marker (nephrin and synaptopodin). (c) Quantification of podocyte markers based on IF staining. (d) Cell death analyzed by TUNEL staining. (e) Cartilage damage evaluated by Safranin O‐Fast Green Staining. (f) Assessment of osteoclasts function by tartrate‐resistant acid phosphatase (TRAP) staining. Data are means ± SEM. *p < 0.05, **p < 0.01, ***p < 0.001; Unpaired Student's *t*‐test was used for statistic analysis.
**Table S1:** Key resource.
**Table S2:** The information of donors
**Table S3:** Primer sequences for qRT‐PCR.

## Data Availability

Single cell transcriptomic data analyzed in this study are publicly available under GEO accession GSE240374. The data that support the findings of this study are available from the corresponding author upon reasonable request.
