# Supplementary material for: Podocyte mPGES‐2 Determines Renal Aging and Contributes to Senile Osteoporosis
Source: Aging Cell. 2026 Jun 25;25(7):e70609. doi: 10.1111/acel.70609 (PMC13296586; doi:10.1111/acel.70609)
Supplement: Supplementary file 1 — Figure S1: Effect of mPGES‐2 deficiency on the morphological and functional changes of multiple tissues. (a) Morphological changes in different tissues assessed by (hematoxylin and eosin) H&E staining. (b) Serum ALT, AST, BUN, and creatinine levels. Data are mean ± SEM. One‐way ANOVA followed by Tukey's test was used for b. O‐KO, old‐knockout; O‐WT, old‐wildtype; Y‐KO, young‐knockout; Y‐WT, young‐wildtype. Figure S2: Effect of mPGES‐2 deficiency on renal damage and podocyte dysfunction caused by aging. (a) Glomerular filtration rate (GFR). (b) The ratio of urine albumin to creatinine. (c) PAS staining of kidney tissues. (d) Quantification of mesangial matrix. (e) Cell death analyzed by TUNEL. (f) Quantification of TUNEL positive staining. (g) Renal fibrosis analyzed by Masson's trichrome staining. (h) Quantification of positive area of Masson's trichrome staining. (i) Renal fibrosis analyzed by Sirius red staining. (j) Quantification of Sirius red positive area. (k) Expression of senescence‐associated secretory phenotype (SASP) factors. Data are presented as means ± SEM. *p < 0.05, **p < 0.01, ***p < 0.001; One‐way ANOVA followed by Tukey's test was used for statistical analysis. Figure S3: Podocyte Ptges2 specific knockout alleviate renal dysfunction and fibrosis caused by aging. (a) Assessment of morphological changes by Periodic acid–Schiff (PAS) staining. (b) Renal fibrosis assayed by Sirius red staining. N‐Cre: Ptges2 f/f ;Nphs2‐Cre. Flox: Ptges2 f/f . Data are means ± SEM. *p < 0.05, **p < 0.01, ***p < 0.001; unpaired Student's t‐test was used for statistical analysis. Figure S4: Tubule Ptges2 specific knockout alleviates renal aging and associated kidney injury. (a) H&E staining. (b) Renal fibrosis assayed by Masson's trichrome staining. (c) Renal fibrosis assayed by Sirius red staining. (d) Cell death analyzed by TUNEL staining. (e) Quantification of TUNEL positive staining. (f) Cell senescence analyzed by β‐Gal staining. (g) mRNA levels of aging biomarkers [file ACEL-25-e70609-s001.docx]

**Podocyte mPGES-2 determines renal aging and contributes to senile osteoporosis**

Dandan Zhong, Chang Hao, Mengyue Li, Jing Liu, Zheng Xu, Jianteng Zhou, Lu Zhao, Siyu Ni, Zhenchao Hu, Yue Sun, Yingying Zou, Dong Sun, Hao Guo, Zhanjun Jia*, Dong Guo*, Jun-Li Cao*, and Ying Sun*

**
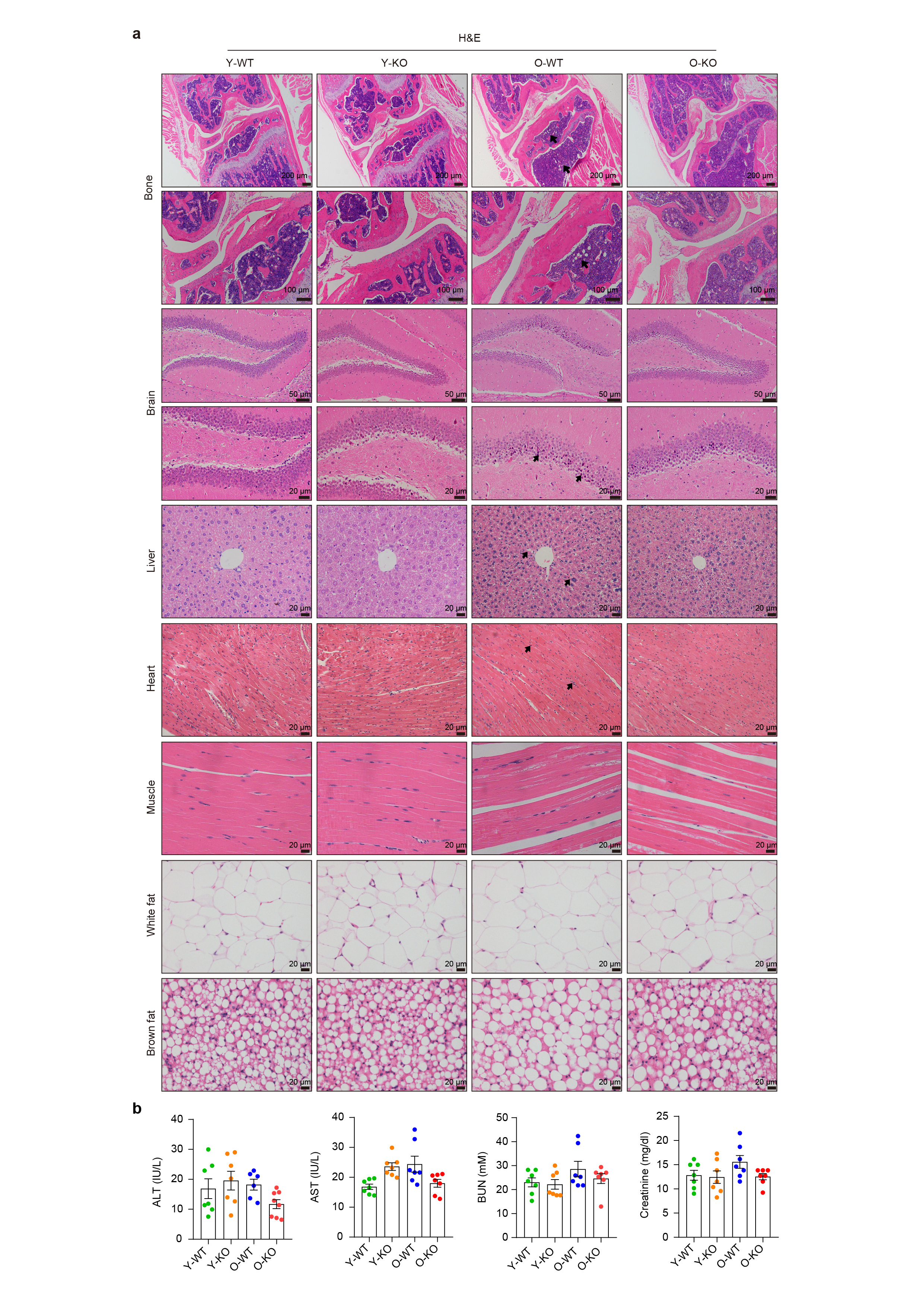
**

**Figure S1.** Effect of mPGES-2 deficiency on the morphological and functional changes of multiple tissues. **a**, Morphological changes in different tissues assessed by (hematoxylin and eosin) H&E staining. **b**, Serum ALT, AST, BUN, and creatinine levels. Data are mean ± SEM. One-way ANOVA followed by Tukey's test was used for **b**. Y-WT: young-wildtype; Y-KO: young-knockout; O-WT: old-wildtype; O-KO: old-knockout.


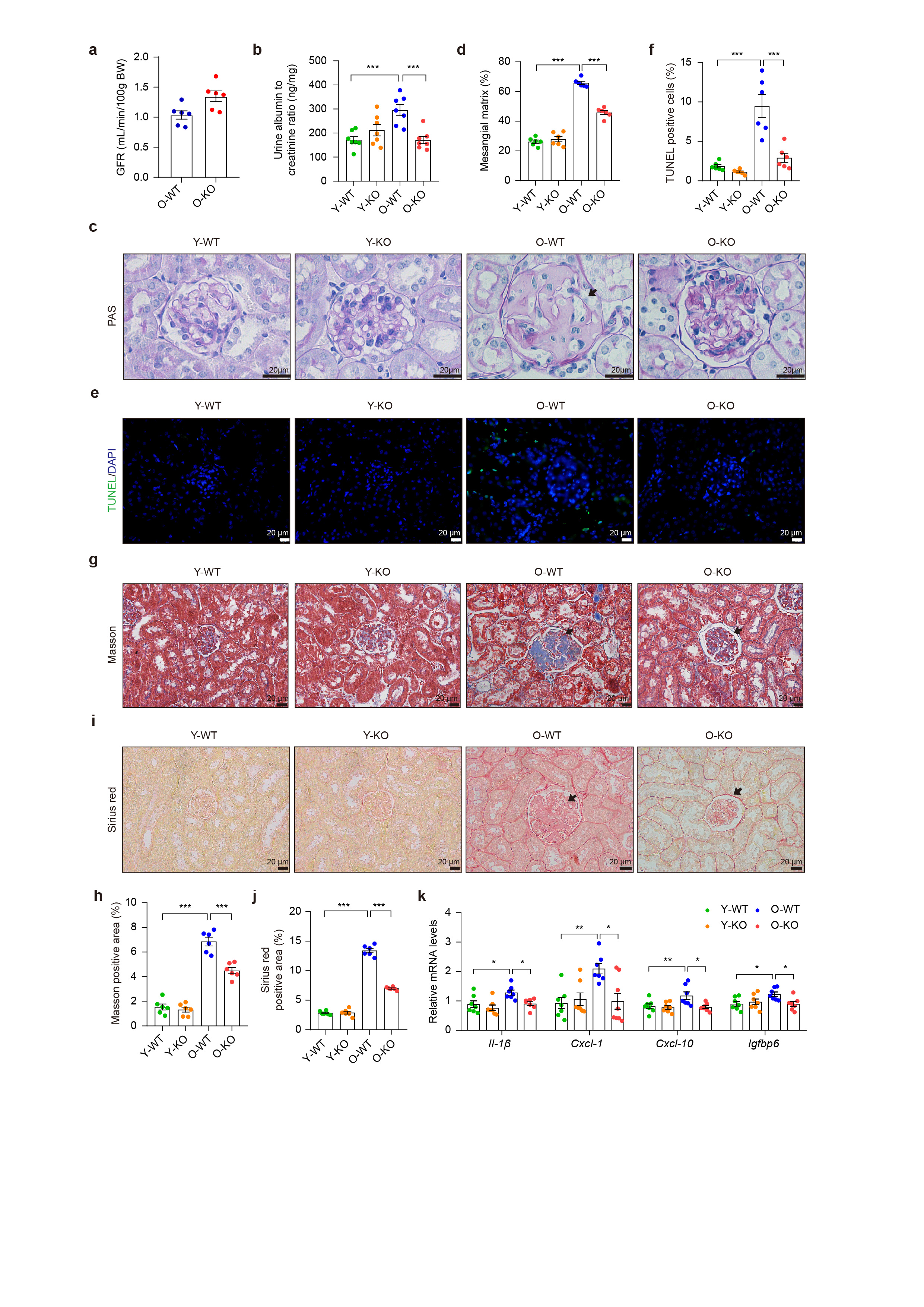


**Figure S2.** Effect of mPGES-2 deficiency on renal damage and podocyte dysfunction caused by aging. **a**, Glomerular filtration rate (GFR). **b**, The ratio of urine albumin to creatinine. **c**, PAS staining of kidney tissues. **d**, Quantification of mesangial matrix. **e**, Cell death analyzed by TUNEL. **f**, Quantification of TUNEL positive staining. **g**, Renal fibrosis analyzed by Masson's trichrome staining. **h**, Quantification of positive area of Masson's trichrome staining. **i**, Renal fibrosis analyzed by Sirius red staining. **j**, Quantification of Sirius red positive area. **k**, Expression of senescence-associated secretory phenotype (SASP) factors. Data are presented as means ± SEM. **P* < 0.05, ***P* < 0.01, ****P* < 0.001; One-way ANOVA followed by Tukey's test was used for statistical analysis.


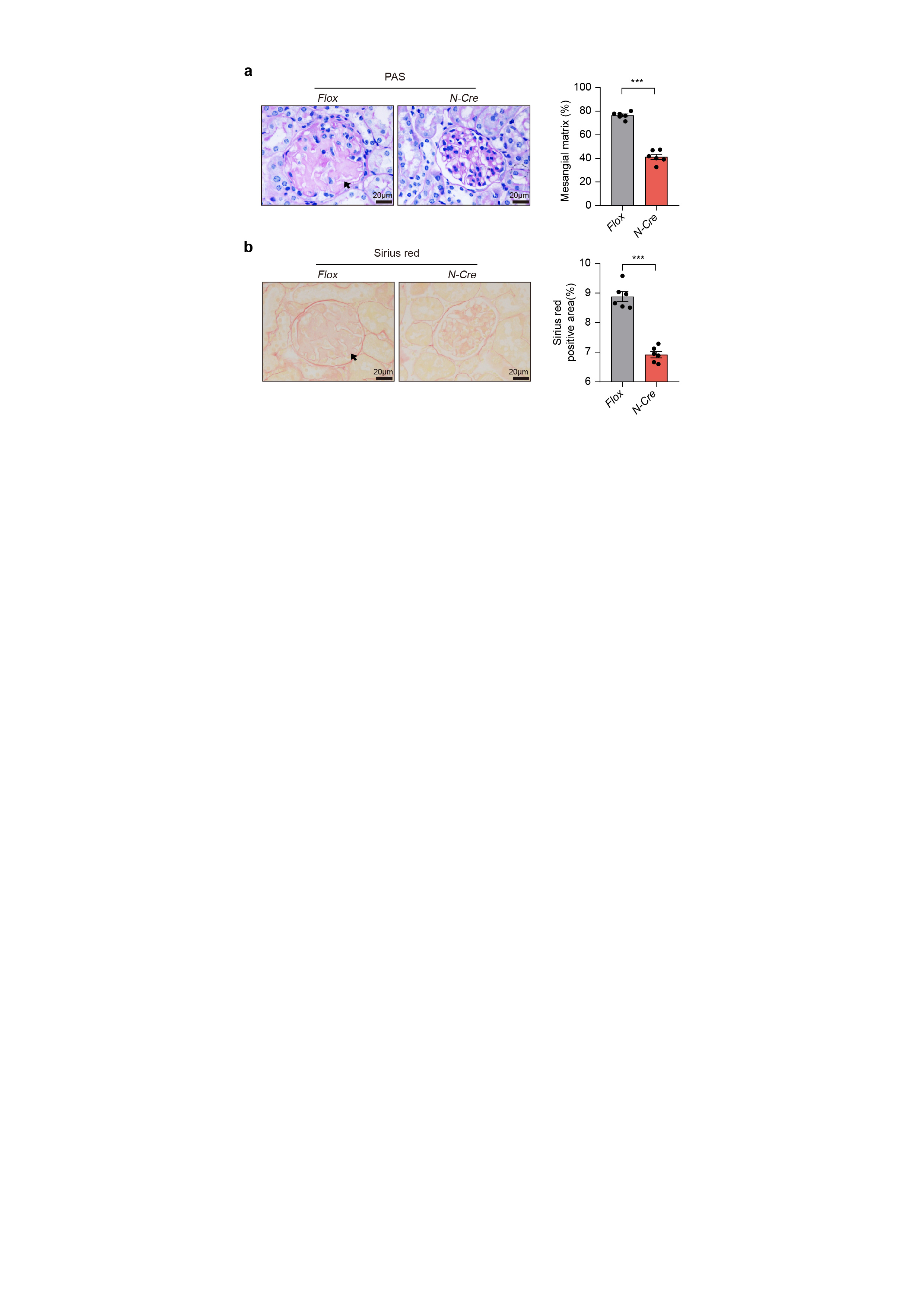
**Figure S3.** Podocyte *Ptges2* specific knockout alleviate renal dysfunction and fibrosis caused by aging. **a**, Assessment of morphological changes by Periodic acid–Schiff (PAS) staining. **b**, Renal fibrosis assayed by Sirius red staining. *N-Cre*: *Ptges2^f/f^;Nphs2-Cre*. *Flox*: *Ptges2^f/f^*. Data are means ± SEM. **P* < 0.05, ***P* < 0.01, ****P* < 0.001; unpaired Student’s *t* test was used for statistical analysis.


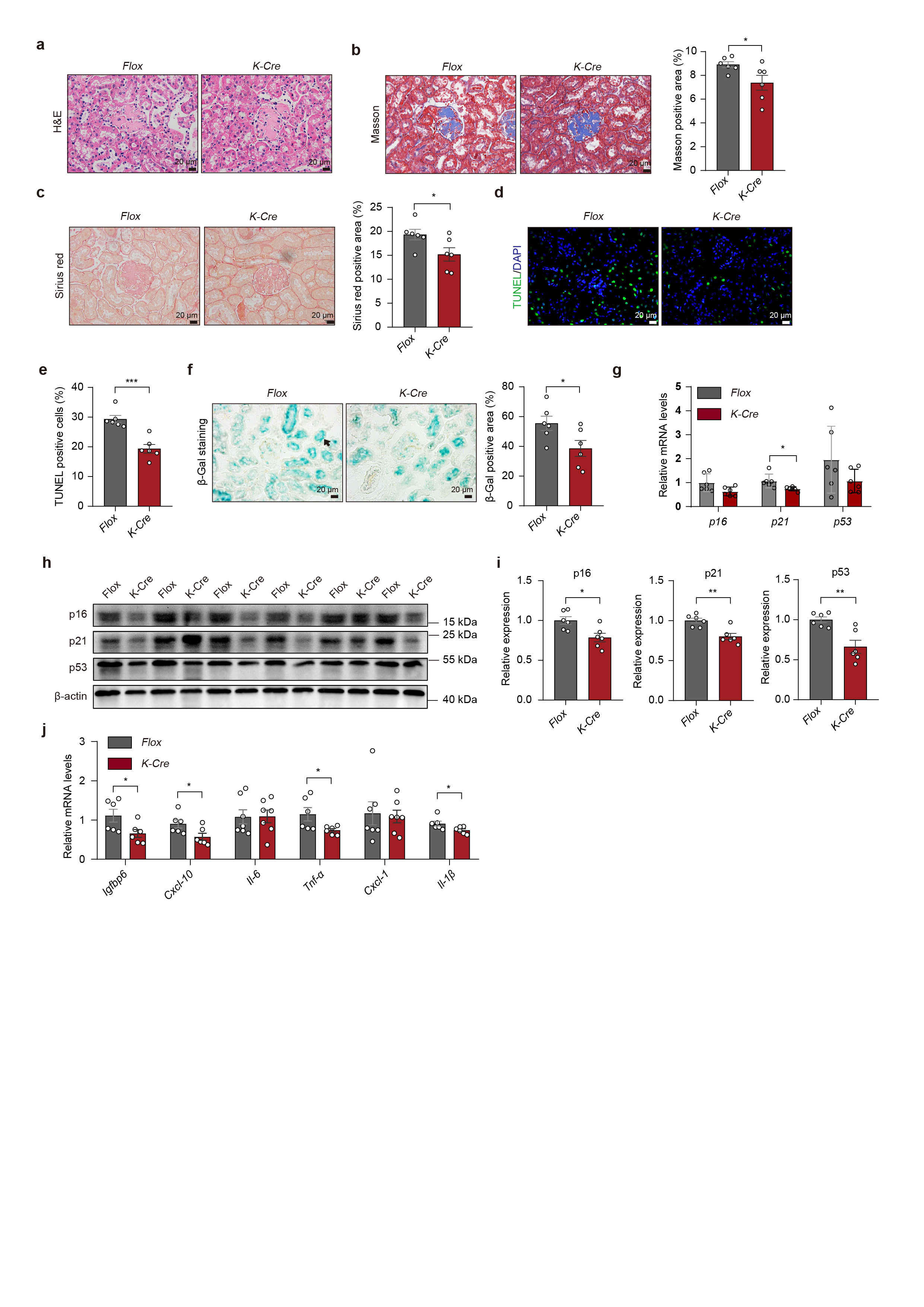


**Figure S4.** Tubule *Ptges2* specific knockout alleviates renal aging and associated kidney injury. **a**, H&E staining. **b**, Renal fibrosis assayed by Masson’s trichrome staining. **c**, Renal fibrosis assayed by Sirius red staining. **d**, Cell death analyzed by TUNEL staining. **e**, Quantification of TUNEL positive staining. **f**, Cell senescence analyzed by β-Gal staining. **g**, mRNA levels of aging biomarkers (p16, p21 and p53). **h**, Representative blots of aging biomarkers of p16, p21and p53. **i**, Quantification of p16, p21 and p53 protein levels. **j**, mRNA levels of SASP factors. *K-Cre*: *Ptges2^f/f^;Ksp-Cre*. *Flox*: *Ptges2^f/f^*. Data are presented as means ± SEM. **P* < 0.05, ***P* < 0.01; Unpaired Student’s *t* test was used for statistical analysis.


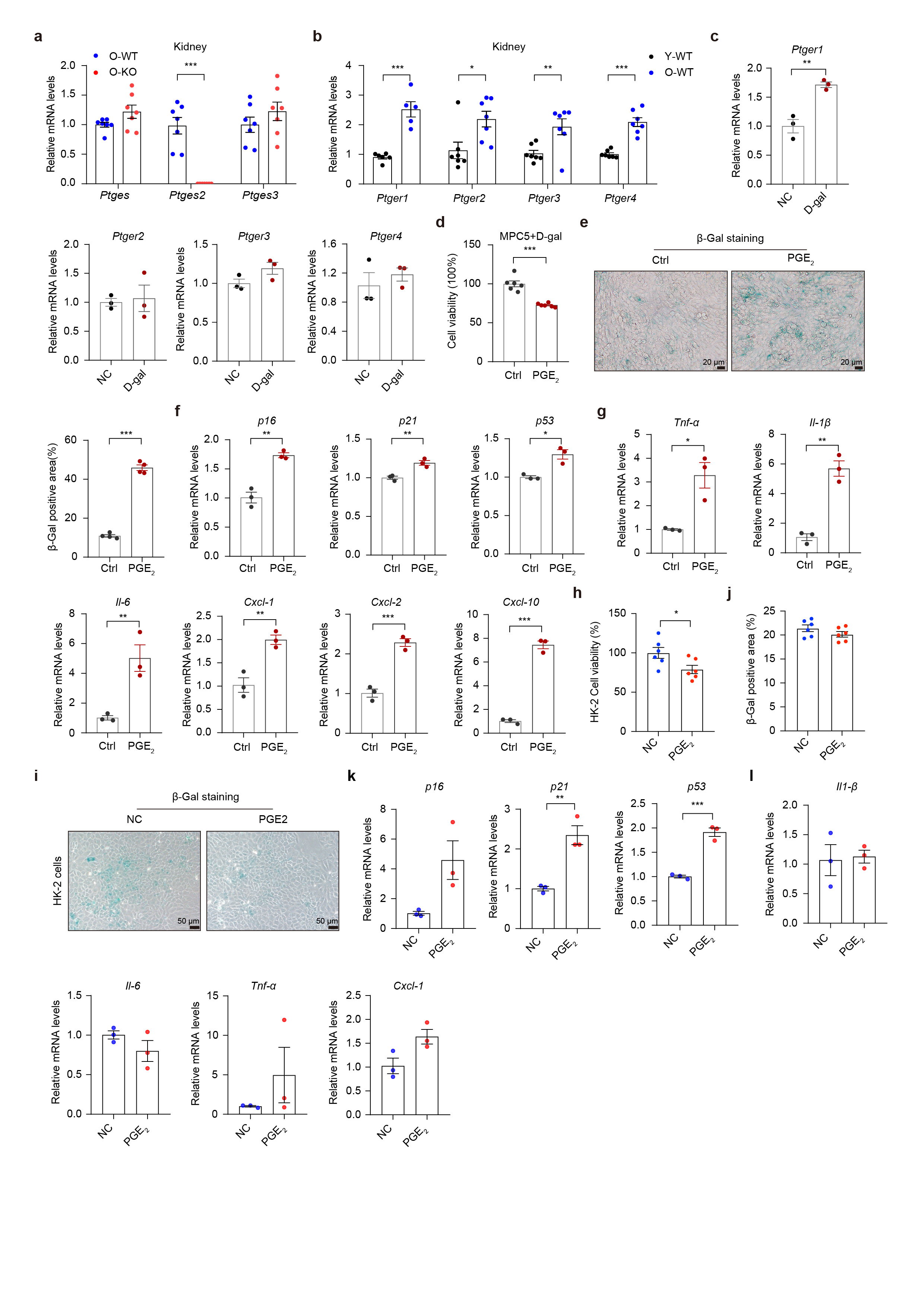


**Figure S5.** PGE_2_ triggers podocyte and tubular cell senescence. **a**, Effect of *Ptges2* knockout on the expression of three known PGE_2_ synthases. **b**, Relative mRNA expression levels of *Ptger*1-4 (EP1-4) in kidneys from young and old wild-type mice. **c**, Relative mRNA expression levels of *Ptger*1-4 in mouse podocyte cells (MPC5) from the control (NC) and D-gal-treated groups. **d**, Effect of PGE_2_ on MPC5 cell viability. **e**, Effect of PGE_2_ on MPC5 cell senescence assayed by β-Gal staining. **f**, Effect of PGE_2_ on mRNA levels of aging biomarkers in MPC5 cells. **g**, Effect of PGE_2_ on mRNA levels of SASP factors in MPC5 cells. **h**, Effect of PGE_2_ on human renal tubular epithelial cells (HK2) cell viability. **i**, β-Gal staining in HK-2 cells. **j**, Quantification of β-Gal positive staining in HK-2 cells. **k**, mRNA levels of senescence markers in HK-2 cells. **l**, SASP factors in HK-2 cells. Data are presented as means ± SEM. **P* < 0.05, ***P* < 0.01, ****P* < 0.001; Unpaired Student’s *t* test was used for statistical analysis.


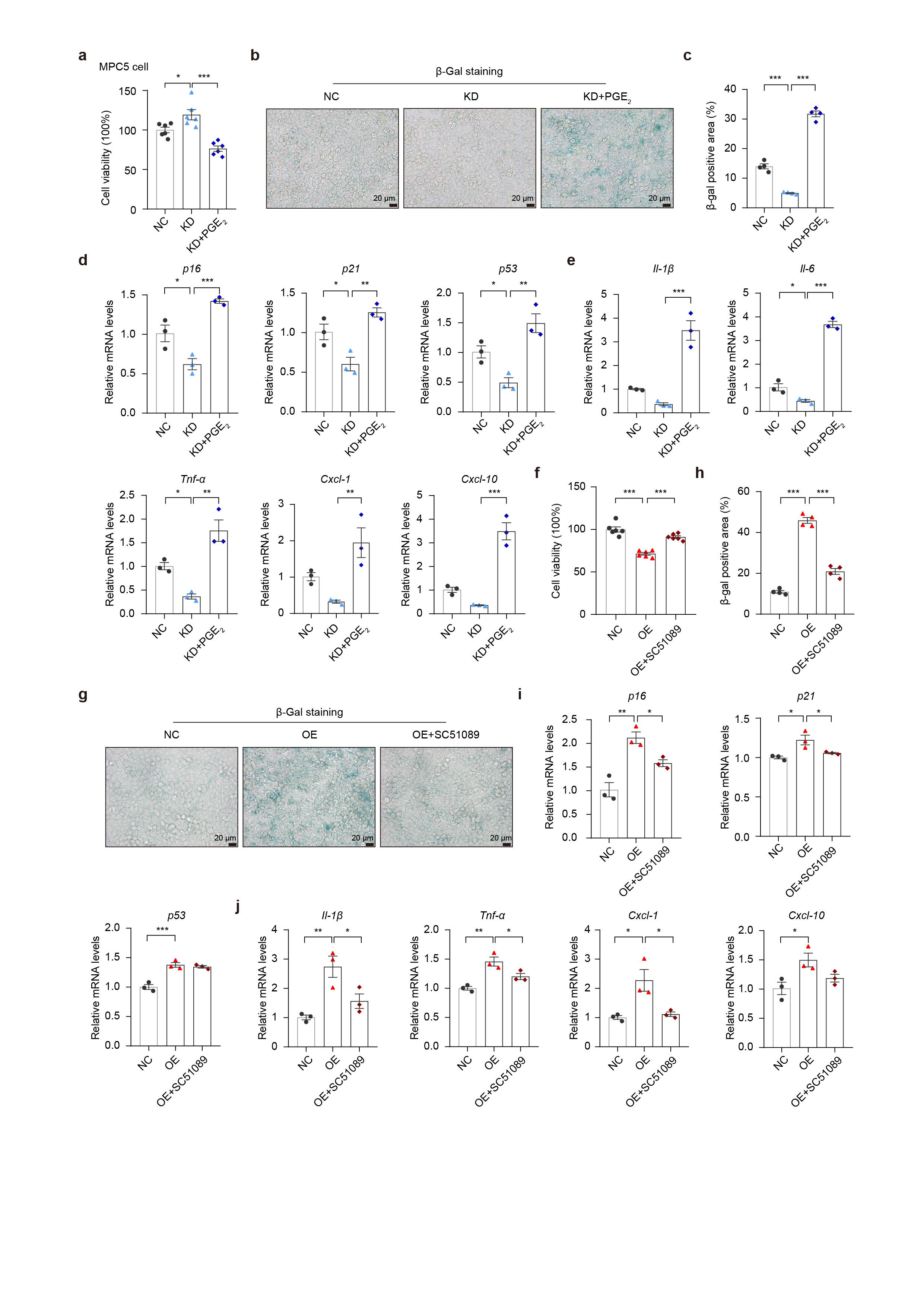


**Figure S6.** Effect of *Ptges2* knockdown and overexpression on podocyte cell senescence. (**a-e**) *Ptges2* knockdown and further exposed to PGE_2_ in MPC5 cells. **a**, Cell viability. **b**, Cell senescence assayed by β-Gal staining. **c**, Quantification of β-Gal positive area. **d**, mRNA levels of aging biomarkers. **e**, mRNA levels of SASP factors. (**f-j**) The effect of *Ptges2* overexpression and further exposed to SC51089, an antagonist of EP1 receptor. **f**, Cell viability. **g**, Cell senescence assayed by β-Gal staining. **h**, Quantification of β-Gal staining. **i**, mRNA levels of aging biomarkers. **j**, mRNA levels of SASP factors. Data are presented as means ± SEM. **P* < 0.05, ***P* < 0.01, ****P* < 0.001; ordinary one-way analysis of variance (ANOVA) with Tukey’s test was used for statistical analysis.


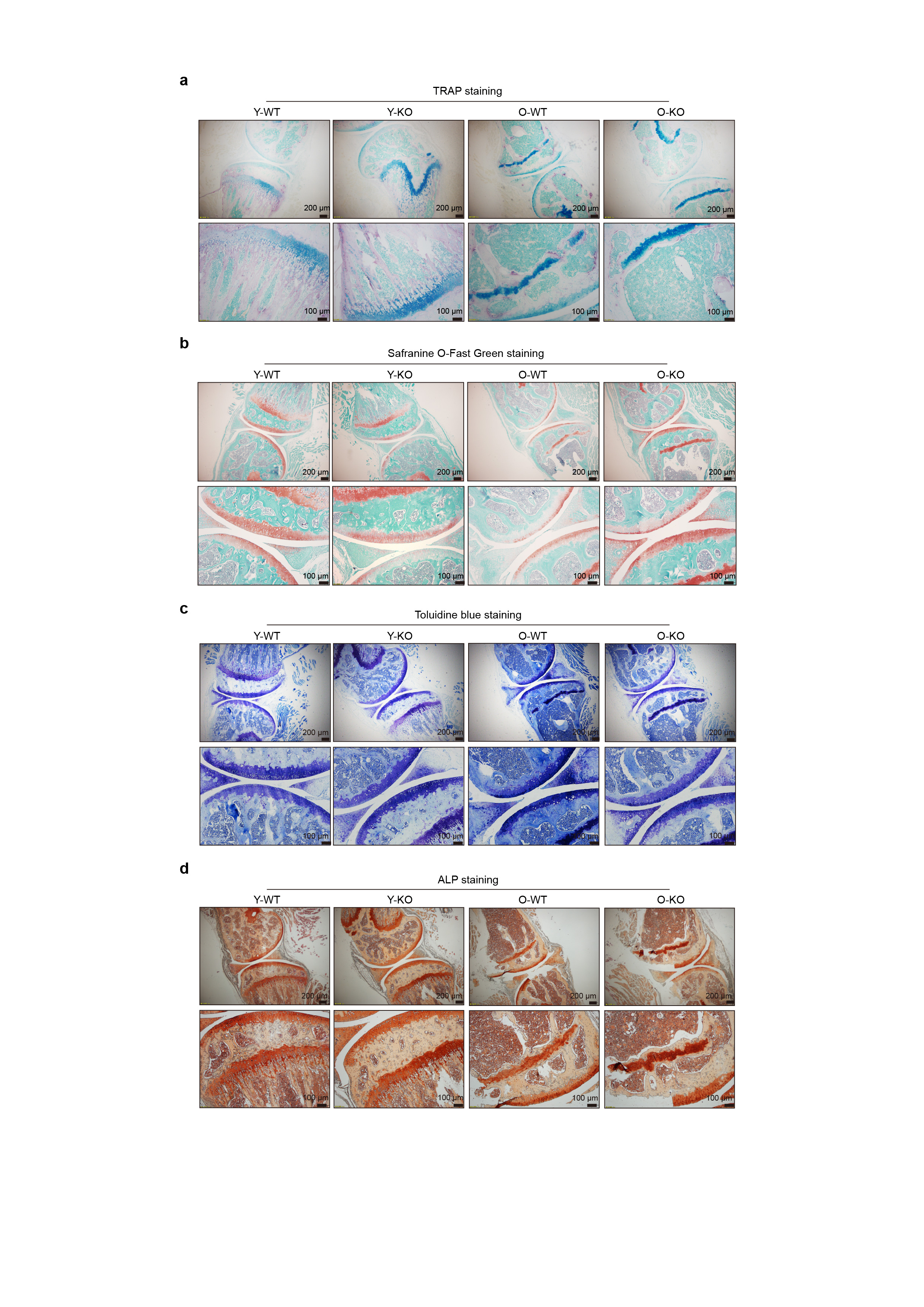


**Figure S7.** Effect of *Ptges2* knockout on bone structure and function. **a**, Assessment of osteoclasts function by tartrate-resistant acid phosphatase (TRAP) staining of *Ptges2* knockout and control mice. **b**, Cartilage damage evaluated by Safranin O-Fast Green Staining. **c**, Bone structural and cellular features assessment by toluidine blue staining. **d**, Assessment of osteoblasts function by ALP staining.


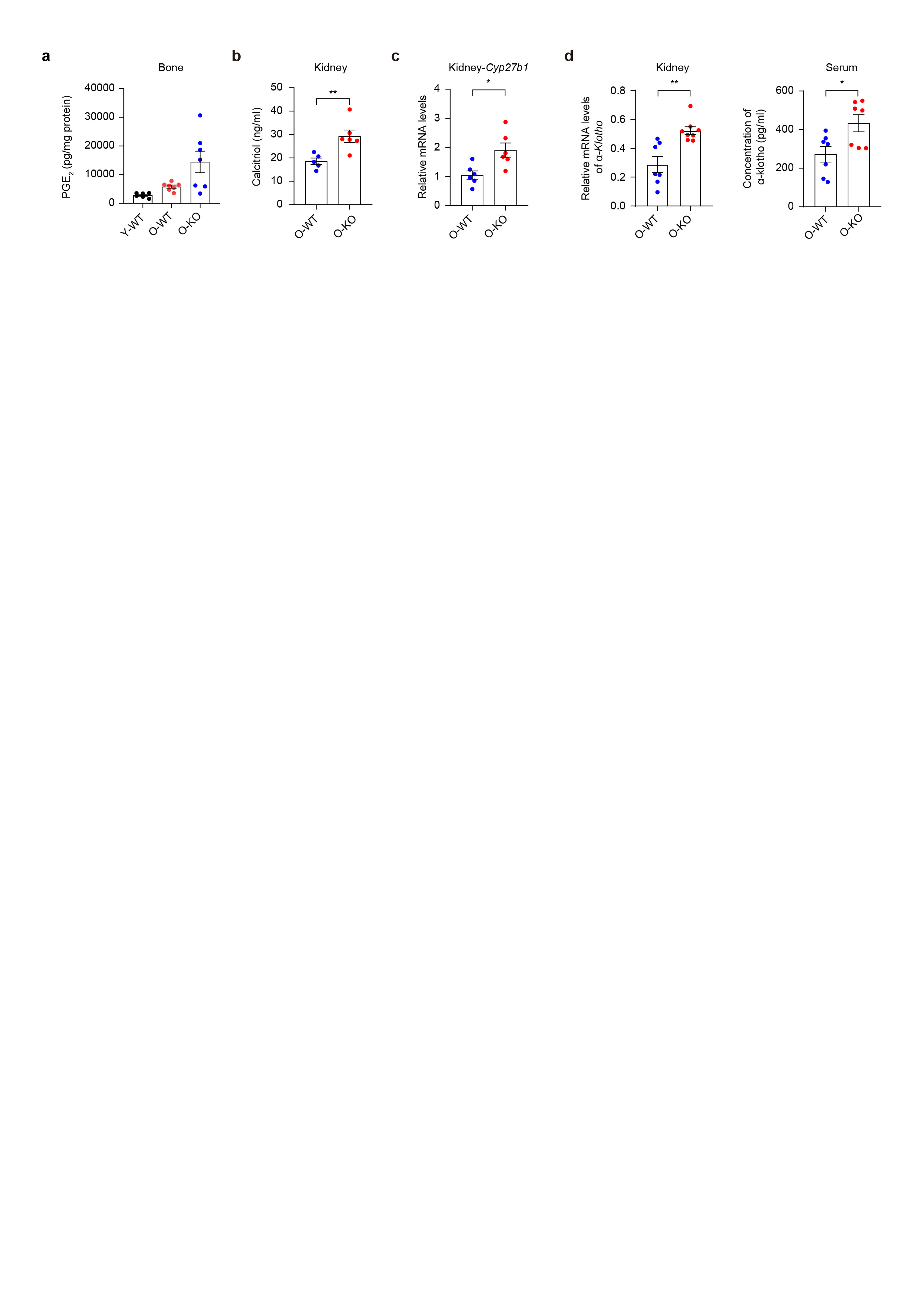


**Figure S8.** Effect of *Ptges2* knockout on hormones associated with bone health. **a**, *Ptges2* knockout on the content of PGE_2_ in the bones. **b**, *Ptges2* knockout on the content of calcitriol, also named as 1,25(OH)_2_D_3_, in the kidneys. **c**, *Ptges2* knockout on the mRNA levels of *Cyp27b1* in the kidneys. **d**, *Ptges2* knockout on the levels of α-klotho in the kidney and serum. Data are presented as mean ± SEM. **P* < 0.05 and ***P* < 0.01; unpaired Student’s *t* test was used for statistical analysis.


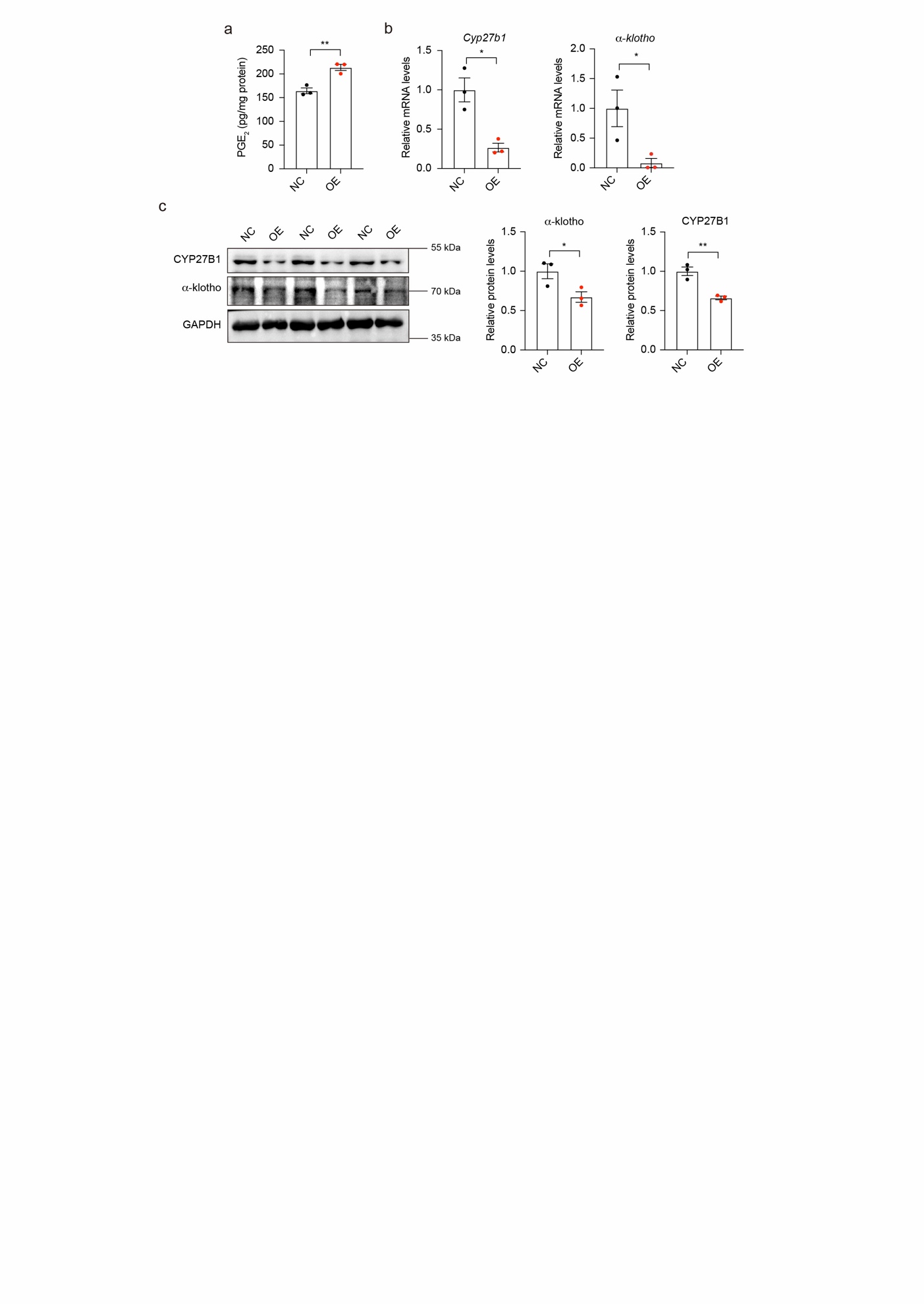


**Figure S9.** Conditioned medium derived from *Ptges2*-overexpressing podocytes suppressed tubular endocrine synthesis capacity. MPC5 podocytes were transfected with a *Ptges2* overexpression plasmid (OE) or the corresponding empty vector (NC). After 24 h, conditioned medium was collected and used to treat HK2 for 48 h. **a**, PGE_2_ levels in conditioned medium collected from MPC5 cells. **b**, Relative mRNA levels of *Cyp27b1* and *α-klotho* in HK2 cells. **c**, Representative western blots and densitometric quantification of CYP27B1 and α-klotho protein levels in HK2 cells. Data are presented as mean ± SEM Statistical significance was assessed using an unpaired two-tailed Student's t-test. **P* < 0.05, ***P* < 0.01.


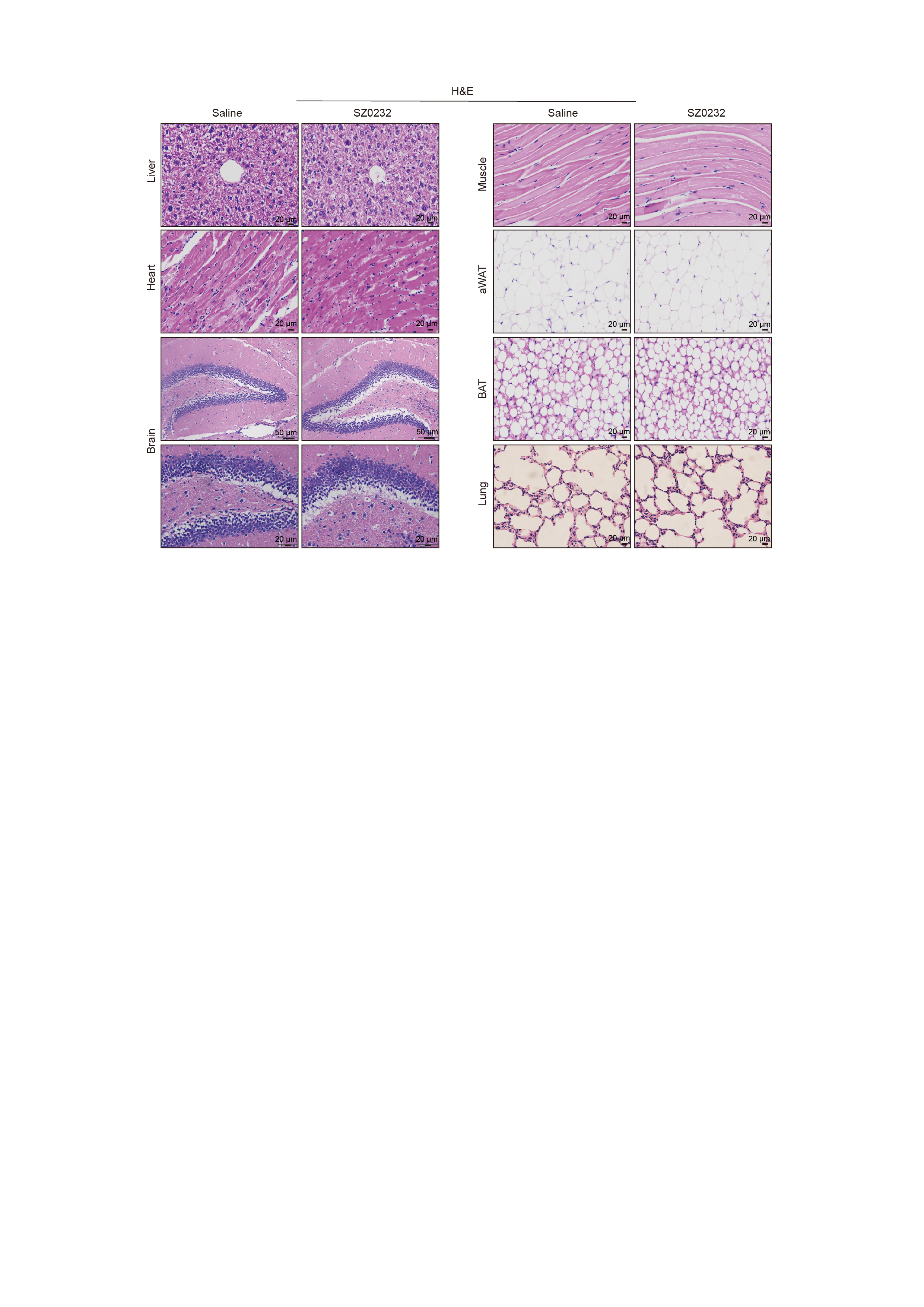


**Figure S10.** Effect of SZ0232 on the morphological changes of multiple tissues at old age. Morphological changes in different tissues from mice treated with SZ0232 or saline assessed by (hematoxylin and eosin) H&E staining.


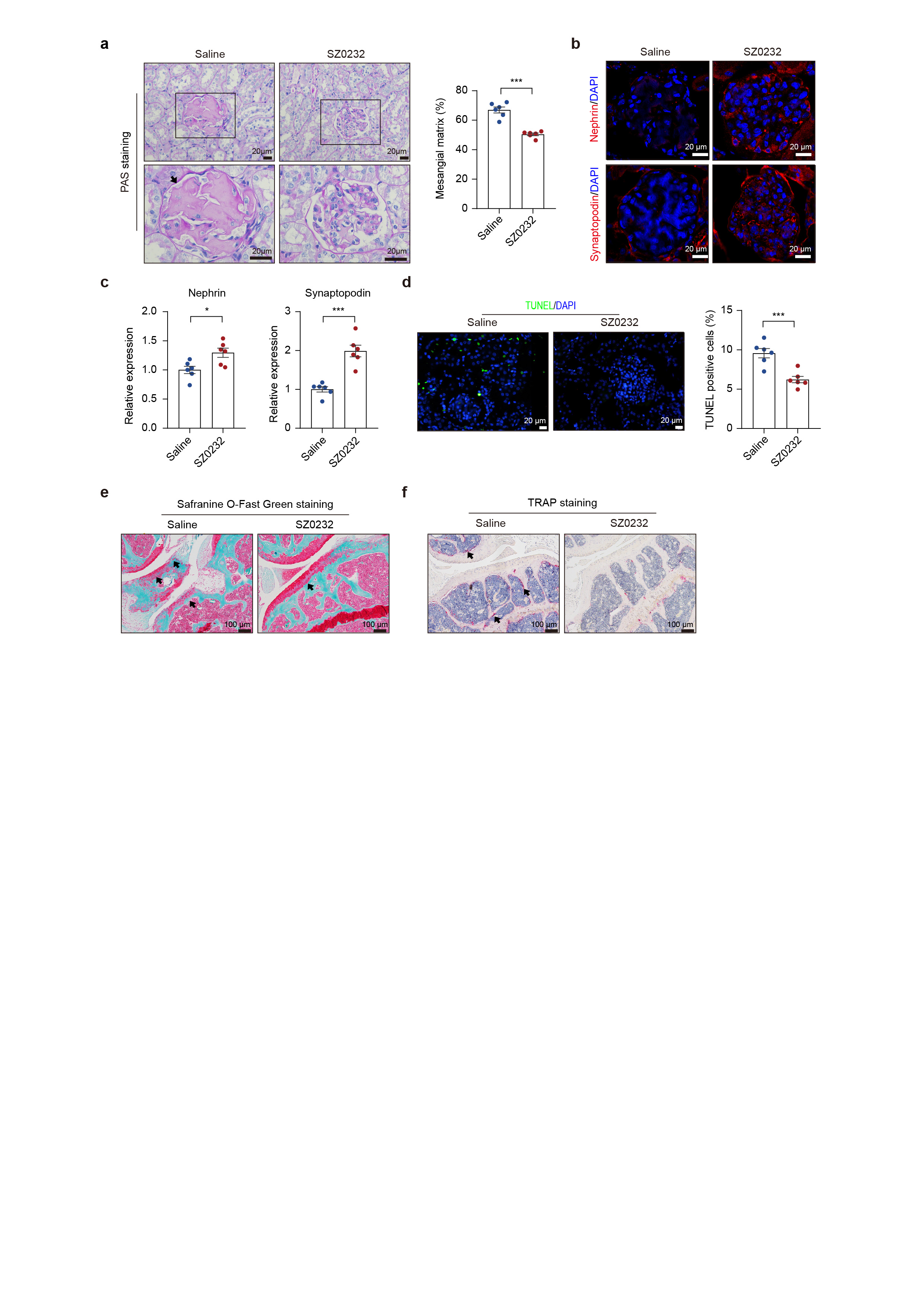


**Figure S11.** mPGES-2 inhibitor SZ0232 protects against aging associated renal and bone dysfunction. **a**, PAS staining of kidney tissues. **b**, Immunofluorescence (IF) staining of podocyte marker (nephrin and synaptopodin). **c**, Quantification of podocyte markers based on IF staining. **d**, Cell death analyzed by TUNEL staining. **e**, Cartilage damage evaluated by Safranin O-Fast Green Staining. **f**, Assessment of osteoclasts function by tartrate-resistant acid phosphatase (TRAP) staining. Data are means ± SEM. **P* < 0.05, ***P* < 0.01, ****P* < 0.001; Unpaired Student’s *t* test was used for statistic analysis.

**Table S1. Key resource**

| REAGENT or RESOURCE | SOURCE | IDENTIFIER |
| --- | --- | --- |
| Antibodies | | |
| Rabbit polyclonal anti-PTGES2 | Proteintech | Cat# 10881-1-AP |
| Monoclonal Mouse Anti--p16 antibody | Santa Cruz | Cat# SC-1661 |
| Monoclonal Mouse Anti--p21 antibody | Santa Cruz | Cat# SC-6246 |
| Monoclonal Mouse Anti--p53 antibody | Santa Cruz | Cat# SC-126 |
| Goat anti-Mouse IgG (H + L) Secondary Antibody, HRP | Beyotime | Cat# A0216 |
| Goat anti-Rabbit IgG (H + L) Secondary Antibody, HRP | Beyotime | Cat# A0208 |
| Rabbit monoclonal anti-Wilms tumor protein | Abcam | Cat#ab89901 |
| Polyclonal Goat anti-Nephrin | R&D systems | Cat# AF3159 |
| Monoclonal Mouse Anti--Synaptopodin | Santa Cruz | Cat# SC-515842 |
| LTL | Thermo Fisher Scientific | Cat#L32480 |
| Donkey Anti-Mouse IgG (H+L) Dylight488 | Earthox | Cat# E032211 |
| Goat Anti-Rabbit IgG (H+L) Dylight488 | Earthox | Cat# E032220 |
| Dylight594 Goat Anti-Mouse IgG (H+L) | Earthox | Cat#E032410 |
| Dylight594 Goat Anti-Rabbit IgG (H+L) | Earthox | Cat# E032420 |
| Chemicals, Peptides, and Recombinant Proteins | | |
| Doxorubicin | Sellek | Cat# S1208 |
| DMEM | Gibco | C11885500BT |
| RPMI 1640 | Thermo Fisher Scientific | C11875500BT |
| FBS | Thermo Fisher Scientific | A5256701 |
| 1X penicillin-streptomycin solution | Beyotime Biotechnology | C0222 |
| Trypsin-EDTA Solution | VICMED | VC2024 |
| D-galactose | [Sigma-Aldrich](https://www.sigmaaldrich.com/) | G0750-25G |
| H2O2 | Sinopharm Chemical Reagent Co., Ltd | 10011208 |
| 16,16-dimethyl Prostaglandin E_2_ | Cayman Chemica | 14750 |
| SC51089 | MedChemExpress | HY-108563 |
| TRIzol reagents | Thermo Fisher Scientific | 15596026CN |
| HyperScript III RT SuperMix | Enzyartisan | R202-02 |
| 2×S6 Universal SYBR qPCR Mix | Enzyartisan | Q204-01 |
| 3% bovine serum albumin | Solarbio | A8020 |
| DAPI | Beyotime Biotechnology | C1002 |
| ECL Plus substrate | eijing Fluorescence Biotechnology Co. Ltd | 044-500 mL |
| Experimental models: Organisms/strains | | |
| C57BL/6J | GemPharmatech LLC. | N/A |
| *Ptges*2^-/-^ mice | GemPharmatech LLC. | T014295 |
| *Ptges2*^flox/-^ mice  *Ksp-Cre* mice  *Nphs*2-*Cre* mice | GemPharmatech LLC.  Peking University  Nanjing Medical University | N/A  N/A  N/A |
| Experimental models: Cell lines | | |
| MPC5  HEK293T | BNCC  ATCC | BNCC342021 CRL-1573 |
| Critical commercial assays | | |
| Masson’s trichrome | Solarbio | G1340 |
| ALP | Nanjing Jiancheng Bioengineering Institute | D001-2-2 |
| TRAP | Servicebio Technology Co., Ltd. | G1050-50T |
| Saffron O and green staining | Solarbio | G1371 |
| Toluidine blue staining | Solarbio | G2543 |
| β-Gal staining | Solarbio | G1580 |
| TUNEL assay | MeilunBio | MA0223-1 |
| Blood urea nitrogen kit | Nanjing Jiancheng Bioengineering Institute | C013-2-1;C011-2-1 |
| Mouse α-Klotho ELISA Kit | Cloud-Clone Corp | SEH757Mu |
| Mouse Urine Albumin Assay Kit | Wuhan Xinqidi Biotech Co., Ltd. | EIA06044m |
| Urine Creatinine Kit | Nanjing Jiancheng Bioengineering Institute | C011-2-1 |
| Prostaglandin E2 ELISA Kit | Cayman Chemical | 514010 |
| Mouse MDA assay kit | Nanjing Jiancheng Bioengineering Institute | A003-4-1 |
| Mouse 1,25-dihydroxyvitamin D3 assay kit | Enovabio | E-HS30655Mo |
| BCA Protein Assay Kit | Thermo Fisher Scientific | 23227 |
| Deposited data | | |
| Source data | This manuscript | N/A |

**Table S2.** The information of donors

| ID | Age (years) | Gender | Source | Applications |
| --- | --- | --- | --- | --- |
| UN003 | 38 | Male | ZK biotech | IHC |
| UN004 | 33 | Male | ZK biotech | IHC |
| UN005 | 27 | Male | ZK biotech | IHC |
| UN006 | 40 | Female | ZK biotech | IHC |
| UN007 | 28 | Male | ZK biotech | IHC |
| UN008 | 40 | Male | ZK biotech | IHC |
| UN009 | 30 | Male | ZK biotech | IHC |
| UN010 | 45 | Male | ZK biotech | IHC |
| UN011 | 35 | Male | ZK biotech | IHC |
| UN012 | 19 | Female | ZK biotech | IHC |
| UN013 | 32 | Male | ZK biotech | IHC |
| UN014 | 50 | Male | ZK biotech | IHC |
| UN015 | 45 | Male | ZK biotech | IHC |
| UN016 | 16 | Male | ZK biotech | IHC |
| UN017 | 17 | Male | ZK biotech | IHC |
| UN018 | 36 | Male | ZK biotech | IHC |
| UN019 | 38 | Female | ZK biotech | IHC |
| UN020 | 36 | Male | ZK biotech | IHC |
| UN021 | 18 | Female | ZK biotech | IHC |
| UN022 | 21 | Male | ZK biotech | IHC |
| UN023 | 17 | Male | ZK biotech | IHC |
| UN024 | 41 | Female | ZK biotech | IHC |
| U-0060 | 67 | Male | ZK biotech | IHC |
| U-0046 | 45 | Female | ZK biotech | IHC |
| U-3476 | 52 | Male | ZK biotech | IHC |
| U-3201 | 47 | Male | ZK biotech | IHC |
| U-3200 | 36 | Female | ZK biotech | IHC |
| U-1029 | 73 | Female | ZK biotech | IHC |
| U-1011 | 57 | Female | ZK biotech | IHC |
| U-1052 | 50 | Male | ZK biotech | IHC |
| U-0030 | 56 | Male | ZK biotech | IHC |
| U-3204 | 39 | Male | ZK biotech | IHC |
| U--0020 | 68 | Female | ZK biotech | IHC |
| U-3203 | 50 | Male | ZK biotech | IHC |
| U-0019 | 56 | Female | ZK biotech | IHC |
| U-3202 | 70 | Male | ZK biotech | IHC |
| U-0017 | 49 | Male | ZK biotech | IHC |
| U-0336 | 72 | Female | ZK biotech | IHC |
| U-0335 | 82 | Male | ZK biotech | IHC |
| U-2093 | 59 | Male | ZK biotech | IHC |
| U-1380 | 54 | Male | ZK biotech | IHC |

**Table S3. Primer sequences for qRT-PCR**

| Gene | Primer sequence (5’→3’) |  |
| --- | --- | --- |
|  | Forward | Reverse |
| Mouse *Ptges2*  Mouse *Ptges1*  Mouse *Ptges3*  Mouse *Ptger1*  Mouse *Ptger2*  Mouse *Ptger3*  Mouse *Ptger4*  Mouse *Beta-actin*  Mouse *p16*  Mouse *p21*  Mouse *p53*  Mouse *Cxcl1*  Mouse *Cxcl2*  Mouse *Cxcl10*  Mouse *Tnf-α*  Mouse *Il6*  Mouse *Il-1β*  Mouse *Alp*  Mouse *Runx2*  Mouse *Opg*  Mouse *Opn*  Mouse *Bmp2*  Mouse *Trap*  Mouse *Ctsk*  Mouse *Igfbp7*  Mouse *Igfbp6*  Mouse *Cyp27b1*  Mouse *α-klotho* | 5’-GCTGGGGCTGTACCACAC-3’  5’-AGCACACTGCTGGTCATCAA-3’  5’-GGTAGAGACCGCCGGAGT-3’  5’-TCCATGACGCTGGGTGCTG-3’  5’-CAAGCTAATGGAGGACTGCAAGAG-3’  5’-GTGTGTGCTGTCCGTCTGTTG-3’  5’-TCTGGTGGTGCTCATCTGCTC-3’  5’-GCTCTGGCTCCTAGCACCAT-3’  5’-TTCAGGTGATGATGATGGGCAACG-3’  5’-CTGGTGATGTCCGACCTGTT-3’  5’-ACCGCCGACCTATCCTTACCATC-3’  5’-GGCTGTTGTGGCCAGTGAA-3’  5’-CCTCCCTCTCATCAGTTCTATGG-3’  5’-AAGTGCTGCCGTCATTTTCT-3’  5’-GAGGATACCACTCCCAACAGACC-3’  5’-AAGTGCATCATCGTTGTTCATACA-3’  5’-TGATACGCCTGAGTGGCTGTCT-3’  5’-TGAGCGACACGGACAAGAAGC-3’  5’-ACTTCGTCAGCATCCTATCAGTTCC-3’  5’-CCCTTGCCCTGACCACTCTTATAC-3’  5’-TGACGATGATGATGACGATGGAGAC-3’  5’-AGCGTCAAGCCAAACACAAACAG-3’  5’-TGCGACCATTGTTAGCCACATACG-3’  5’-CAGCAGAACGGAGGCATTGAC-3’  5’-GCGAGCAAGGGTCTCTGATA-3’  5’-GCAGAAGAATCCACGGACCT-3’  5’-ACCAGCGTGCTTGCGGATTG-3’  5’-TGACTTTGTGCTAGGCTGGTTTG-3’ | 5’-GATTCACCTCCACCACCTGA-3’  5’-CTCCACATCTGGGTCACTCC-3’  5’-TCGTACCACTTTGCAGAAGCA-3’  5’-GCGACGAACAACAGGAAGGTG-3’  5’-CAGTGCCAGTGCGATGAGATTC-3’  5’-CTTCTCCTTTCCCATCTGTGTCTTG-3’  5’-ATCTGGGTTTCTGCTGATGTCTTTC-3’  5’-GCCACCGATCCACACAGAGT-3’  5’-CGGGCGGGAGAAGGTAGTGG-3’  5’-TCAAAGTTCCACCGTTCTCG-3’  5’-GGCACAAACACGAACCTCAAAGC-3’  5’-CGCCCTTGAGAGTGGCTATG-3’  5’-CGTGGGCTACAGGCTTGTC-3’  5’-GTGGCAATGATCTCAACACG-3’  5’-AAGTGCATCATCGTTGTTCATACA-3’  5’-TTCTCCACAGCCACAATGAG-3’  5’-CACAAGAGCAGTGAGCGCTGAA-3’  5’-CCTGGTAGTTGTTGTGAGCGTAATC-3’  5’-CGTCAGCGTCAACACCATCATTC-3’  5’-CCCTTCCTCACACTCACACACTC-3’  5’-TGTAGGGACGATTGGAGTGAAAGTG-3’  5’-AGGTGCCACGATCCAGTCATTC-3’  5’-GCACATAGCCCACACCGTTCTC-3’  5’-ACCGCCAGGCAGATTGAGG-3’  5’-AAGAACACCTTGGCACCAGT-3’  5’-GGAACGACACTGCTGCTTTC-3’  5’-GCTTGTGGCCGGAGGAGGAG-3’  5’-AATCAGGCAGAAGAGACGAGAGG-3’ |
